# Ectopic expression of miRNA172 in tomato (*Solanum lycopersicum*) reveals novel function in fruit development through regulation of an *AP2* transcription factor

**DOI:** 10.1186/s12870-020-02489-y

**Published:** 2020-06-19

**Authors:** Mi-Young Chung, Ujjal Kumar Nath, Julia Vrebalov, Nigel Gapper, Je Min Lee, Do-Jin Lee, Chang Kil Kim, James Giovannoni

**Affiliations:** 1 Department of Agricultural Education, Sunchon National University, Suncheon, South Korea; 2 Department of Genetics and Plant Breeding, Bangladesh Agricultural University, Mymensingh, 2202 Bangladesh; 3 Boyce Thompson Institute for Plant Research, Cornell University, Ithaca, New York, USA; 4 Department of Horticulture, Kyungpook National University, Daegu, Korea; 5 US Department of Agriculture/Agriculture Research Service, Robert W. Holley Centre for Agriculture and Health, Ithaca, New York, USA

**Keywords:** Tomato, *SlAP2a*, miR172, Fruit ripening, Ethylene

## Abstract

**Background:**

MicroRNAs (miRNAs) are short non-coding RNAs that can influence gene expression via diverse mechanisms. Tomato is a fruit widely consumed for its flavor, culinary attributes, and high nutritional quality. Tomato fruit are climacteric and fleshy, and their ripening is regulated by endogenous and exogenous signals operating through a coordinated genetic network. Much research has been conducted on mechanisms of tomato fruit ripening, but the roles of miRNA-regulated repression/expression of specific regulatory genes are not well documented.

**Results:**

In this study, we demonstrate that miR172 specifically targets four *SlAP2* transcription factor genes in tomato. Among them, *SlAP2a* was repressed by the overexpression of *SlmiR172*, manifesting in altered flower morphology, development and accelerated ripening. *miR172* over-expression lines specifically repressed *SlAP2a*, enhancing ethylene biosynthesis, fruit color and additional ripening characteristics. Most previously described ripening-regulatory genes, including *RIN*-*MADS*, *NR*, *TAGL1* and *LeHB*-*1* were not influenced by miR172 while *CNR* showed altered expression.

**Conclusions:**

Tomato fruit ripening is directly influenced by miR172 targeting of the *APETALA2* transcription factor, *SlAP2a*, with minimal influence over additional known ripening-regulatory genes. miR172a-guided *SlAP2a* expression provides insight into another layer of genetic control of ripening and a target for modifying the quality and nutritional value of tomato and possibly other fleshy fruit crops.

## Background

MicroRNAs (miRNAs) are short, non-coding RNAs of approximately 18–24 nucleotides in length. Eukaryotic miRNAs derive from endogenously-processed RNA transcripts as long, primary transcripts (pri-miRNA), which are then transcribed by a ribonuclease III-like nuclease and Dicer-like (DCL) proteins. Among the four DCL proteins in *Arabidopsis thaliana*, DCL1 participates in the production of miRNAs with the aid of other accessory proteins [1, 2]. The long pri-miRNAs adopt imperfect single-stranded hairpin structures that contain the miRNA in one arm of the stem [3]. This folding is essential for their processing by RNAse III, which cleaves them into pre-miRNAs. The miRNAs are broadly grouped as intronic (transcribed from the intron of the host transcript) and intergenic (transcribed independently by DNA-dependent RNA polymerase II) [4].

miRNAs function as developmentally regulated repressors of gene expression either by mediating the cleavage of complementary mRNA or by suppressing translation [5–10]. Mature miRNAs can be incorporated into the RNA-induced silencing complex (RISC) or other RISC-like complexes involved in the negative regulation of target genes [6, 11–15]. During rapid changes of developmental phases [16], one strand of the miRNA duplex is abandoned by unraveling or cleavage [17], and the other strand is retained in the RISC for recognition of target mRNA. Transcriptional silencing by targeted cleavage has been found to predominate in plants, facilitating straight-forward predictions of likely target genes using computational approaches based on available genome sequences [18–21].

In plants, miRNAs are involved in environmental interactions, numerous developmental processes (for example, the control of meristem identity, cell proliferation, developmental timing, and pattern formation events), and serve as key regulators of organ development [15, 22]. At the post-transcriptional level, plant miRNAs have been shown to regulate metabolic processes via binding *cis*- and *trans*-regulatory sites to repress protein translation [21, 23–25]. The role of miRNAs in regulating gene expression has been extensively investigated regarding growth and development, signal transduction, hormone signaling, innate immunity, and response to diverse biotic and abiotic stresses in *Arabidopsis* and other plant species [15, 26–28].

Fleshy fruit ripening is the summation of numerous biochemical and physiological processes coordinated by developmental, hormonal, and environmental cues influencing pigmentation, texture, aroma, flavor, and nutritional composition [29]. Tomato (*Solanum lycopersicum*) is the most studied model for fleshy fruit because of its simple diploid genetics and small genome size (900 Mb) [30], short life cycle, efficient transformation, distinct phenotypes in ripening, dense genetic maps, expanding genomic resources, and the availability of its high-quality genome sequence [31–33].

Positional cloning of well-characterized tomato mutants, such as *rin* (*ripening-inhibitor*), *Gr* (*green-ripe*), *Nr* (*never-ripe*)*, gf* (*green flesh*), and *Cnr* (*colorless non-ripening*), provided insights into fundamental aspects of molecular regulation in fruit ripening, most notably, the ripening-regulatory pathway influencing ethylene biosynthesis and signaling [29, 34–37]. Additional regulators have been identified by transcriptome analysis, and their roles confirmed through either repression, overexpression, or *CRISPR/Cas9* gene editing (*NAC1*, *FUL1*, *FUL2*, *MADS1*, *TAGL1*, and *SlAP2a*) [38–42], or interaction with other regulators (*LeHB-1* and *TDR4*) [42–44]. Potential regulatory networks of miRNAs and the *RIN-MADS* gene during tomato fruit development and ripening have been elucidated through deep sequencing and expression profiling of the identified miRNAs in the wild-type (WT) tomato [45]. miRNAs have additionally been associated with DNA methylation and promoter activity [46].

The miR172 family has been implicated in the regulation of a subfamily of *APETALA2* (*AP2*) transcription factor genes [47, 48]. In *Arabidopsis*, miR172 influences floral organ identity through *AP2* [49], and flowering time through down-regulation of *TOE1* (*TARGET OF EAT1*) and *TOE2* (*TARGET OF EAT2*) [50]. In maize, miR172 has been found to block the binding site of the *AP2* family gene *glossy15* (*gl15*) and plays a central role in maintaining juvenility [51]. Although miR156 regulates the juvenile-to-adult phase transition in *Arabidopsis* and delays flowering, as evident from the overexpression of miR156, the opposite phenotype was found in overexpressed miR172 lines [52–54].

Genome sequences contribute to miRNA discovery. Yin et al. [55] detected 21 conserved miRNAs and their corresponding target genes in tomato, including five putative *AP2* family targets of miR172, by adopting a computational homology search approach. To further address the role of miRNAs in fruit biology, we characterized the functional roles of the *AP2* family genes and their miRNA regulation. Specifically, we analyzed the role of miR172 in floral determination and fruit ripening in tomato and show that miR172 overexpression mimics *SlAP2a* RNAi repression phenotypes. We further demonstrated a regulatory role of miR172 operating through *SlAP2a* in fleshy fruit development distinct from activities previously identified in the dry fruit of *Arabidopsis*.

## Results

### Functional analysis of miR172 in tomato

**Fig. 1:**
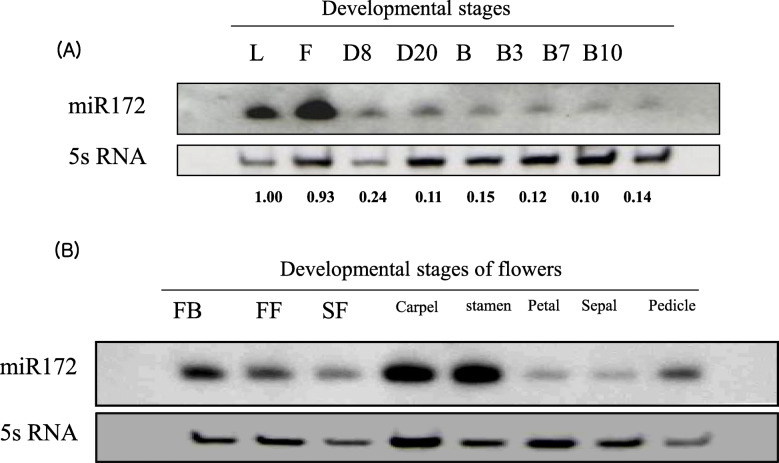
Expression pattern of miR172 in wild-type tomato (cv. Ailsa Craig) at different developmental stages. **a** Northern blot analysis of miR172 from leaf, flower, and fruit at different development stages: D8 (8 days after 1 cm fruit), D20 (20 days after 1 cm fruit), B (breaker), B3 (3 days after breaker), B7 (7 days after breaker), B10 (10 days after breaker); **b** Northern blot analysis of miR172 from flower organs at different developmental stages: FB (flower bud), FF (full flower), SF (senescence flower)

Accumulation of miR172 in tomato was assessed by RNA gel-blot analysis. Figure 1a and additional file 1 show that miR172 accumulated predominantly in leaves and flowers and declined during fruit development. The miR172 transcript accumulated abundantly during the early stages of flower bud, carpel, and stamen development (Fig. 1b and additional file 1).

**Fig. 2:**
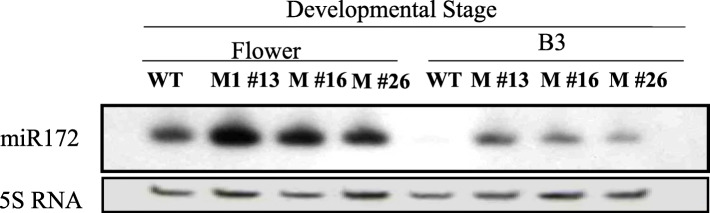
RNA gel blot analysis of miR172 levels in wild-type (WT) and *mir172* OE lines (M #13, M #16, M #26) at B3 (3 days after breaker)

To analyze the function of miR172 in tomato*,* 403 bp of genomic sequence spanning miR172 (pre-miR172; Tomato Bacterial Artificial Chromosome (BAC)-end seq_*Mbo*I (SL_*Mbo*I0036L22_T7_268826)/Tomato WGS Chromosomes SL2.40ch11) (solgenomics.net), termed *SlmiR172a* (NCBI accession 102,464,333), was amplified by PCR from the indicated BAC clone. This sequence was ligated downstream of the cauliflower mosaic virus (*CaMV*) 35S promoter in vector pBI121, transformed into *Agrobacterium tumefaciens* strain LBA4404 and transformed the WT tomato cv. Ailsa Craig. Twenty-five *kanamycin* (*kan*) resistant independent transformed lines (T_0_) were confirmed by PCR of the *kan* gene. Three of these overexpression (OE) lines were selected based on phenotype and copy number (two strong phenotypes lines 13 and 16, and one weak single-copy phenotype line 26) in the T_2_ generation and used for subsequent studies. The miR172 transcript was abundant in the three homozygous transgenic lines compared with WT controls in both flowers and 3 days post-breaker stage (early ripening) fruit, with higher abundance in flowers than B3 fruit (Fig. 2 and additional file 1).

### Overexpression of miR172 influences flowering time and floral pattern in tomato

**Fig. 3:**
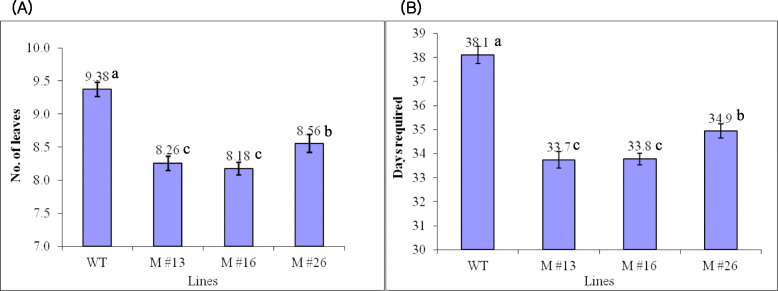
Flowering times in wild-type (WT) and three *miR172* OE lines (M #13, M #16, M #26). **a** Number of leaves formed before first inflorescence; **b** Number of days required for first flowering from sowing in a total of 25 flowers from WT and three different *miR172* OE lines. Mean comparison by Tukey test at *p <* 0.05

**Fig. 4:**
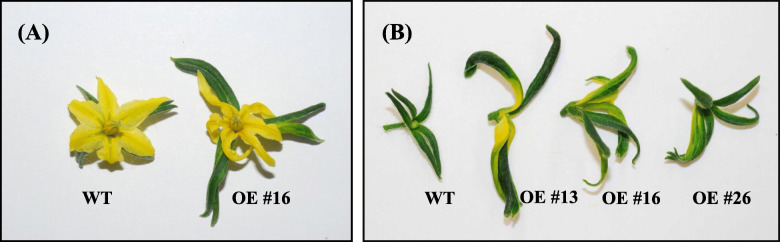
Phenotypes of flowers in wild-type and *miR172*OE lines (T_2_ generation). Transgenic flower that overexpressed miR172 showed enlarged sepal and more curved, rippled petal compared with the wild-type; **b** Flowers with removed petals and stamen; *miR172* overexpression lines showed a range of sepal-to-petal transformations depending on expression level

miR172 is known to affect floral organ identity and flowering time via translational repression of *AP2* family members in *Arabidopsis* [49, 50, 56]. The number of leaves produced before the first inflorescence in *SlmiR172a* OE lines was, on average, one leaf less than in the WT when grown in 16-h light/8-h dark photoperiod (tomato is day-neutral; Fig. 3a). The time to first flower was reduced by 4–5 days in *miR172* OE lines (Fig. 3b). In addition, overexpression of *SlmiR172* resulted in enlarged sepals, narrow petals, and sepal-to-petal transformation (Fig. 4a, b).

### SlmiR172a regulates fruit ripening through SlAP2a

**Fig. 5:**
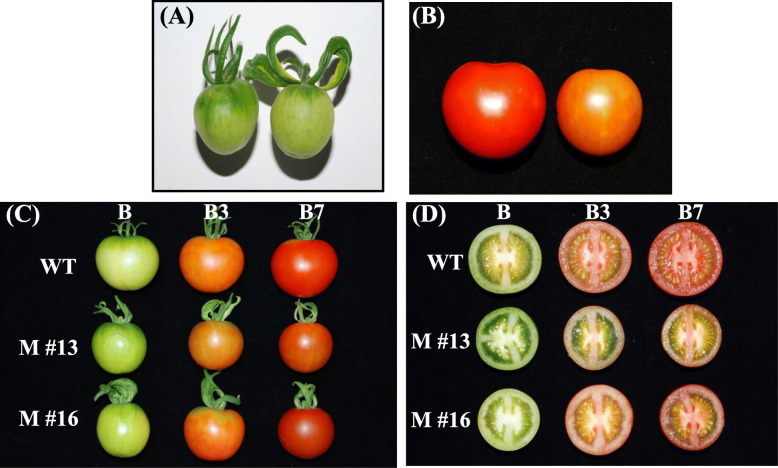
Phenotypes of fruit in wild-type and three different *miR172* OE lines. **a** Immature fruit (8 days after 1 cm fruit) of wild-type (left) and *miR172* OE lines (right); **b** Late stage of fruit ripening (10 days after breaker) in wild-type (left) showed normal pattern of ripening, but fruit in *miR172* OE line (right) did not change to full red color; **c** Fruits in wild-type and *miR172* OE lines showed normal pattern of ripening at different developmental stages (B, B3, B7) but fruit in *miR172* OE line #13 did not change to full red color; **d** Transverse section of wild-type fruit and *miR172* OE lines at different stages of fruit ripening (B, breaker; B3, 3 days after breaker; B7, 7 days after breaker)

Repression of *SlAP2a* by miRNA in tomato affected fruit ripening, ethylene production, ripening time, carotenoid biosynthesis, and the expression of ripening-related genes [42, 57]. *SlAP2a* has been shown to contain a putative *SlmiR172a* binding site [42]. Here, the duration of development from 1 cm fruit to breaker in *SlmiR172a* OE lines was 4–6 days earlier than in untransformed controls (Table 1), resembling the effect of *SlAP2a* repression. At early fruit development (8 days after 1 cm), an enlarged calyx was apparent (Fig. 5a). No distinct differences in ripe fruit color intensity were noted between the *SlmiR172a* OE lines and WT (Fig. 5).

**Table 1:** Days required for fruit maturation as measured from 1 cm fruit to breaker stage in wild-type and *miR172* OE lines

Genotype	Days^a^
Wild-type	28.43 ± 0.14 a
*miR172* OE #13	22.54 ± 0.12 c
*miR172* OE #16	23.18 ± 0.17 c
*miR172* OE #26	24.52 ± 0.19 b

^a^Values represent mean ± SE of at least 10 fruit from each line; lowercase letters (a, b, c) denote significant difference by Tukey test at *p <* 0.05

**Fig. 6:**
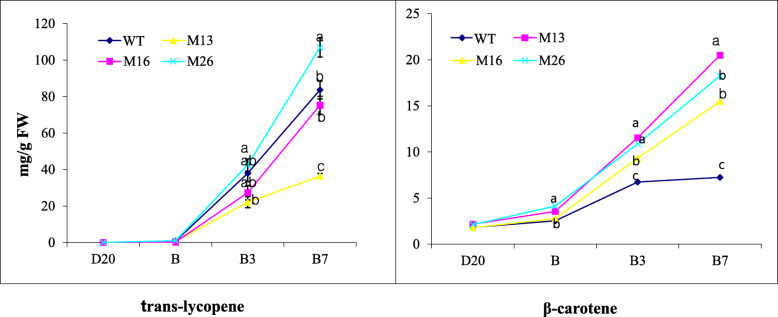
*Trans-*lycopene and β-carotene content of wild-type (cv. Ailsa Craig) and three different *miR172* OE lines during tomato fruit ripening. *Trans*-lycopene and β-carotene contents of three different *miR172* OE lines and wild-type fruits were quantified by HPLC at different developmental stages (D20, B, B3, and B7, as defined in the Methods). Error bars represent SE of three replicates. FW, fresh weight. Mean comparison by Tukey test at *p <* 0.05

**Fig. 7:**
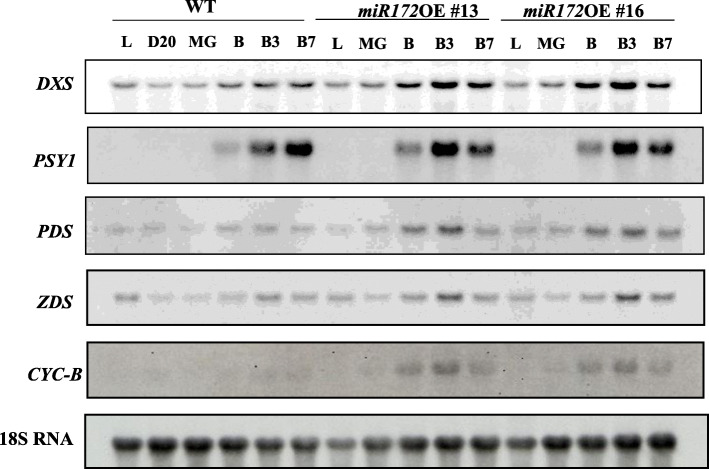
Expression of genes related to carotenoid biosynthesis in wild-type (WT) and *miR172* OE lines (#13 and #16). Total RNA was extracted from fruit at various developmental stages (L, D20, B, B3, and B7, as defined in the Methods). Twenty micrograms of RNA was loaded and hybridized with *DXS*, *PSY1*, *PDS*, *ZDS*, and *CYC-B* gene-specific probes. The filter-strip rehybridized with tomato 18S RNA probe was used as a control

For precise characterization of pigment composition in *SlmiR172a* OE and WT lines, we measured carotenoid of ripe fruit using HPLC. Pericarp lycopene accumulation in *SlmiR172a* OE lines M #16 and M #26 was similar to WT, but dramatically reduced in line M #13 (Fig. 6). β-carotene was elevated approximately two-fold in the fruit of all three *SlmiR172a* OE lines relative to WT. We also measured expression of carotenoid biosynthesis genes including *DXS1* (*1-deoxy-D-xylulose 5-phosphate synthase*), *PSY1* (*phytoene synthase 1*), *PDS* (*phytoene desaturase*), and *ZDS* (*ζ-carotene desaturase*), which showed abundant mRNA accumulation, and only the downstream gene *CYC-B* (*lycopene-β-cyclase*) was substantially increased in *SlmiR172a* OE lines compared to the WT (Fig. 7 and additional file 1). *CYC-B* catalyzes the conversion of lycopene to β*-*carotene, which was significantly elevated in the ripe fruit of *SlmiR172a* OE lines consistent with elevated *CYC-B* expression.

### Function of miR172 in regulation of ethylene production

**Fig. 8:**
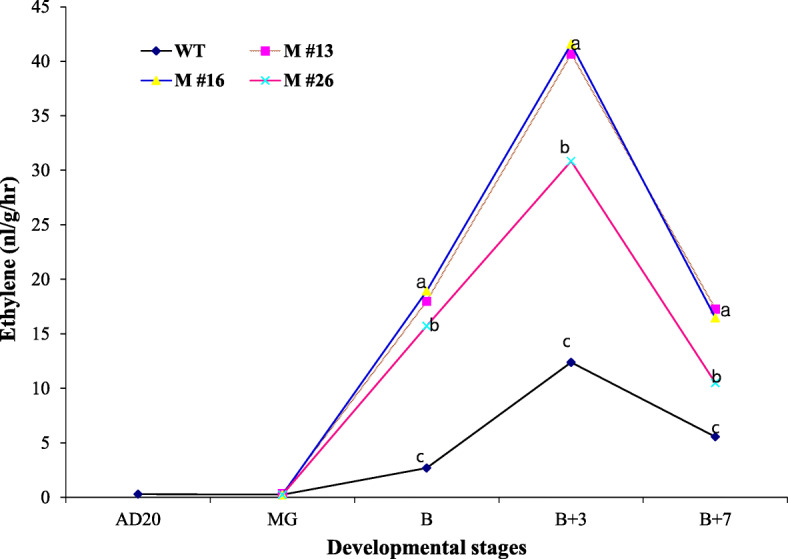
Production of ethylene in the fruits of wild-type (WT) and three different *miR172 OE* lines (#13, #16, and #26) at different developmental stages: D20 (20 days after 1 cm fruit), MG (24 days after 1 cm fruit), B (breaker), B3 (3 days after breaker), B7 (7 days after breaker). Values represent mean of at least 15 individual fruit. Mean comparison by Tukey test at *p <* 0.05

Ethylene is a critical ripening hormone of climacteric fruit, including tomato. To determine the role of miR172 on fruit ethylene production, we measured ethylene evolution at five fruit development stages by gas chromatography. *miR172a* OE fruit ethylene increased three- to four-fold from onset of ripening compared to the WT control, and remained unchanged 7 days after breaker (Fig. 8).

**Fig. 9:**
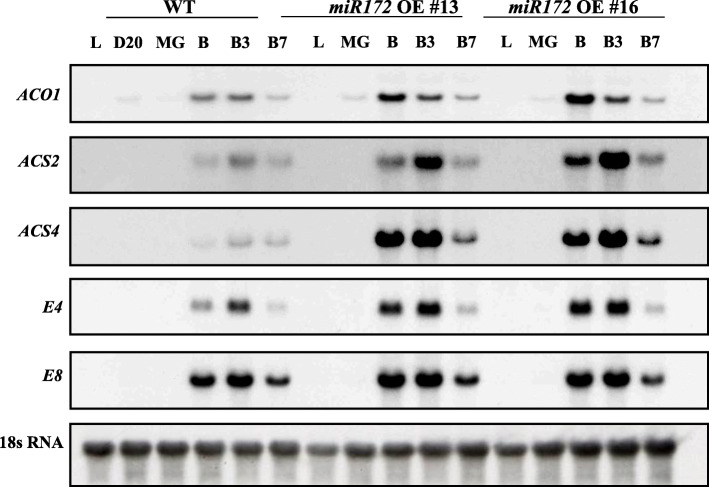
Gene expression of ethylene biosynthesis-related genes *ACO1*, *ACS2*, and *ACS4*, as well as the level of *E4* and *E8* in wild-type and *miR172* OE lines (#13 and #16). Total RNA was extracted from fruit at various developmental stages (D20, B, B3, and B7), as well as from leaf. Twenty micrograms of RNA was used in northern blot with P32-labeled cDNA probes. The filter-stripped and rehybridized tomato 18S RNA probe was used as a control

We monitored mRNA abundance of ethylene biosynthesis genes, *1-AMINOCYCLOPROPANE 1-CARBOXYLIC ACID SYNTHASE* (*ACS2*), *ACS4*, and *1-AMINOCYCLOPROPANE-1-CARBOXYLIC ACID OXIDASE* (*ACO1*), and ethylene-responsive (*E4* and *E8*) genes during fruit development. All five genes were induced approximately five-fold higher in *miR172a* OE lines at B and B3 stages of fruit ripening as compared to WT (Fig. 9 and additional file 1). Taken together, ethylene and corresponding gene expression data indicate that *miR172a* influences fruit ethylene biosynthesis through repression of the *SlAP2a* transcription factor. *miR172a* OE phenocopied *SlAP2a* repression [42].

### Identification of miR172-guided RNA cleavage and its role in miR172a OE lines

**Fig. 10:**
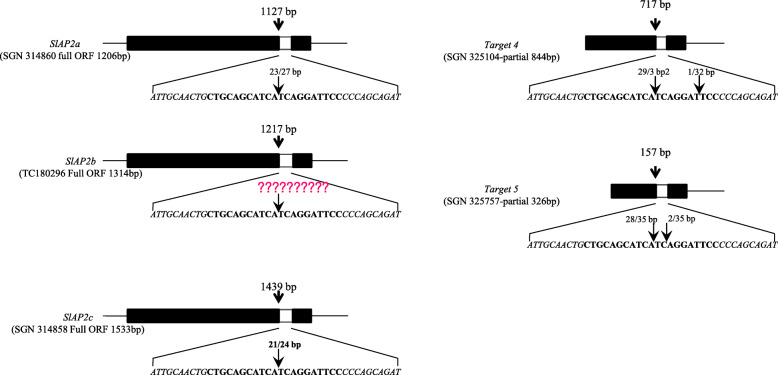
Mapping of miR172-mediated mRNA cleavage sites by RNA ligase 5′-RACE PCR. Thick black block represents open reading frames (ORFs), and white block represents putative miRNA interaction site, with expended region in bold text. Arrows in the expanded sequence indicate the 5′-RACE clones corresponding to each site, and the positions inferred as 5′-ends of miRNA-guided cleavage products

To predict miR172-guided cleavage products, we aligned miR172 and five putative target gene sequences *SlAP2a*, *SlAP2b*, *SlAP2c*, *Target 4*, and *Target 5* (Supplementary data 1). We then used RNA ligase-mediated 5′-rapid amplification of cDNA ends (5′-RACE) on five predicted targets of *miR172a*. Four out of five predicted targets showed specific cleavage sites that corresponded to the miRNA complementary sequence, except for *SlAP2b* (Fig. 10). All cleavage events occurred close to the middle of the complementary strands, identical to the cleavage site of *Arabidopsis* miR172. *SlAP2b* did not show cleavage (Fig. 10). We attempted to detect cleavage of *SlAP2b* using repeated RACE-PCR with many additional 3′-gene-specific primers to no avail though it is possible technical issues could be the cause. However, RACE-PCR with a 19-bp adapter sequence and 5-bp cleavage sequence primers amplified target sequence in *miR172a* OE lines.

We performed RT-PCR to assess transcript abundance of predicted target genes in *miR172* OE lines and WT and observed amplification of *SlAP2a* and *SlAP2* at B3 and *AP2b* in flower of *miR172a* OE lines (Supplementary Fig. S1A,B and additional file 1). Meanwhile, the cleavage products of *Target 4* and *Target 5* genes amplified in WT and *miR172a* OE lines at B3. Only *SlAP2a* was detected by northern blot in B and B3 *miR172a* OE fruit (Supplementary Fig. S2 and additional file 1). Other *SlAP2* (*SlAP2b* and *SlAP2c*) genes were not detected, consistent with *SlAP2a’s* known role in fruit development and ripening. Accumulation of the *SlAP2a* transcript was reduced in *miR172a* OE lines compared to WT, indicating that tomato *miR172a* is involved in down-regulating *SlAP2* genes (*SlAP2a*, *SlAP2b*, and *SlAP2c*) via miRNA binding site cleavage.

### miR172 and ripening-related regulatory genes

**Fig. 11:**
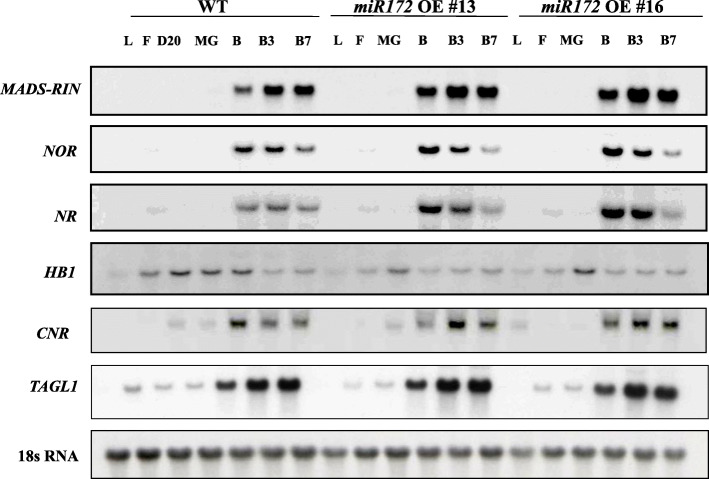
Expression of ripening-related genes in wild-type and *miR172* OE lines. Total RNA was extracted from at various developmental stages (L, F, D20, B, B3, and B7, as defined in the Methods). A total of 20 μg of RNA was used in northern blot with P32-labeled *RIN*, *NOR*, *NR*, *HB1*, *CNR*, and *TAGL1* cDNA probes. The filter-stripped and rehybridized tomato 18S rRNA probe was used as a control

The observed role of *miR172a* in fruit ripening and floral organ identity motivated us to investigate the relationship if any between *miR172* and other ripening regulatory genes, specifically, *MADS-RIN* (*RIPENING INHIBITOR*), *CNR-SPB* (*COLORLESS NON-RIPENING*), *HB1* (*HD-ZIP HOMEOBOX PROTEIN-1*), and *NOR* (*NON-RIPENING*). During fruit ripening, only *NR* showed expression differences between *miR172* OE lines and WT (Fig. 11 and additional file 1). We additionally performed RNA gel-blot analysis of miR172 in several tissues of genotypes harboring mutations in these ripening genes (*rin*, *nor*, *Cnr*, and *Nr*; Supplementary Fig. S3 and additional file 1). Only Cnr showed miR172 levels that were different than WT with miR172 transcript higher in ripe *Cnr* compared to WT (Supplementary Fig. S4). This result suggests that *SlCNR* is influenced by miR172 though this effect may be indirect and possibly mediated by *SlAP2a*.

## Discussion

miR172 is a highly conserved miRNAs in plants. miR156/157, miR170/171, and miR165/166 have also evolved as orthologs. Together these miRNAs play important and conserved roles in plant and especially floral development [15, 20, 48, 58–60]. For example, miR172, miR156, and miR159 influence flowering time in *A. thaliana* [50, 61, 62]. Three different tomato miR172 sequences were identified via sRNA cloning from leaf, flower bud, and fruit [61, 62], with the loop structure precursors denoted miR172a, miR172b, and miR172c [58, 63]. Although the tomato genome sequence was published in 2012 [64], investigations of miRNAs in tomato are limited. In this study, we annotated the pre-miRNA sequence (pre-miR172; Tomato BAC-end seq_*Mbo*I (SL_*Mbo*I0036L22_T7_268826)/Tomato WGS Chromosomes SL2.40ch11) as *SlmiR172a*, which is similar to the predicted sequence reported by Itaya et al. [61], and overexpressed it under the 35S promoter. The transgenic plants affected not only flowering in *Arabidopsis* but also fruit ripening presumably through interaction with its likely target gene, *SlAP2a* [43].

### Floral homeotic transformation and early flowering in miR172 OE lines

The miR172 transcript was abundant at the early stages of tomato flower bud, carpel, and stamen development (Fig. 1b), similar to C-group floral homeotic genes [50, 65, 66]. This temporal expression pattern of miR172 is similar to *Arabidopsis* where accumulation gradually increases shortly after germination until flowering [50, 67]. Expression of tomato miR172 is consistent with function in floral and fruit development, presumably as in floral organ identity in *Arabidopsis* and *Antirrhinum* via regulation of genes acting in the ABC floral development model [66, 68]. In the ABC model, transcription factor genes comprise three classes of floral homeotic genes. Here we demonstrate that overexpression of *SlmiR172a* in tomato affected floral pattern as manifested by enlarged sepals and sepal-to-petal transformation (Fig. 4). Additional phenotypes correlated with the transcriptional abundance of *SlmiR172a* (Fig. 1a, b and additional file 1). Interestingly, tomato sepal-to-petal transformation differed from phenotypes observed with *Arabidopsis* miR172 overexpression, in which accumulation of 35S driven miR172 expression resulted in a phenotype similar to *Arabidopsis ap2* mutants including abnormal and late-flowering [49, 69]. Furthermore, overexpression of miR172-resistant *AP2* resulted in elevated AP2 protein and presented a defective floral pattern resembling the *ag* (*agamous*) mutant, including loss of floral determinacy, suggesting miR172 down-regulates *AP2* during floral development [49, 70]. *Arabidopsis* sepal-to-petal transformation was similarly observed with overexpression of the B function genes *PISTILLATA* (*PI*) [71, 72] in addition to *GREEN PETAL* in petunia [73]. Floral development phenotypes of tomato *ap2* mutants beyond *SlAP2a*’s role in ripening have not been reported to date. Thus, we cannot conclusively state that floral shift with overexpression of *SlmiR172a* in tomato result from repression of *SlAP2* genes. Interestingly, tomato *TAP3* and *SlGLO1*, B function genes shown to specify petal and stamen identities in their respective mutants, were also characterized by conversion of petals to sepal-like structures as well as conversion of stamens to carpel-like organs [74–76].

Previously reported activation–tagging showed *miR172*’s involvement in *Arabidopsis* floral development and flowering-time control via post-transcriptional repression of two *AP2*-like genes, *TOE1* and *TOE2* [48, 50, 77, 78]. miR172 is influenced by *GIGANTEA* (*GI*) but not *CONSTANS* (*CO*) though *CO* is regulated by *GI* in addition to *phytochromes* (*PHY*s) and *cryptochromes* (*CRY*s). *GI*-mediated miR172 influences flowering time by regulating *FLOWERING LOCUS T* (*FT*) and *TOE1* [67, 79, 80]. Overexpression of *SlmiR172a* in tomato induced flowering 4–5 days earlier than WT or approximately one leaf earlier in phylotaxy (Fig. 3a, b). In transgenic tomato, the *SFT* (*SINGLE FLOWER TRUSS*) gene has been shown to induce flowering earlier than in the WT [81, 82], and the *sft* mutant delayed flowering time. Considering these data, we assumed that *SlmiR172* and its target genes control the genetic pathway of flowering mediated by *SFT* expression in photoperiod-insensitive tomato, as like photoperiodic *Arabidopsis*. Previously, we reported *SlAP2b* and *SlAP2c* genes with a *SlmiR172*-specific perfect complementary binding site (Supplementary data 1), though no phenotypic changes with repression of *SlAP2b* and *SlAP2c* either in the floral specification or flowering time were noted. As such, these phenotypes resulting from overexpression of *SlmiR172a* may reflect functional redundancy of targeted genes.

### Overexpression of SlmiR172a phenocopies ripening effects of SlAP2a repression

We predicted computationaly that miRNA172 targets the *AP2* gene family. Previous studies in *Arabidopsis*, rose, and maize verified interaction of miR172 and *AP2* family genes [48–50, 53, 83, 84]. We previously demonstrated elevated *SlAP2a* transcript levels at the onset of fruit ripening, but not in flowers, and a role in ethylene regulation and ripening [42]. Like *AP2* family genes in *Arabidopsi*s and maize, *SlAP2a* also contains a miR172 binding site.

Ripening tomato fruits accumulate carotenoids, mainly lycopene and β-carotene, via gene expression-regulated metabolic flux influenced by the *PSY1*, *PDS*, *ZDS*, and *CYC-B* genes [85, 86]. *SlAP2a* repression resulted in reduced lycopene and elevated β-carotene manifesting in orange fruit via reduced *PSY1* and elevated *CYC-B* expression [42]. Fruit pigmentation in *SlmiR172a* OE lines was consistent with *SlAP2a* RNAi [42, 57, 62, 87] in that beta-carotene was substantially elevated. Indeed, *SlmiR172a* OE line #13 had identical pigmentation to *SlAP2a* RNAi and the strongest transgenic expression line had greatest reduction in lycopene content (Figs. 5, 6, 7). When investigating the intensity of phenotypic traits influenced by miRNAs, miRNA accumulation has been shown to correlate with miRNA overexpression [88, 89].

We further demonstrated that *SlmiR172a* overexpression suppressed *SlAP2a* mRNA accumulation and *SlmiR172a* OE lines produced 3–4 times more ethylene during fruit ripening than WT consistent with increased expression of ethylene biosynthesis genes *ACS2* and *ACS4* (Figs. 8 and 9). This enhanced ethylene was identical to that observed in *SlAP2a* RNAi lines [42]. Fruit ripening time was 4–5 days earlier in *SlmiR172a* OE than WT, again similar to *SlAP2a* RNAi (Table 1). Ripening-acceleration in response to *SlmiR172a* OE has similarities to miR172 regulation of *GLOSSY15* (*GL15*) in maize and *Arabidopsis* in that *GL15* gene plays a primary role in maintaining juvenility while miR172 induction represses GL15 to promote developmental transitioning [51, 83, 90].

### Identification of miR172-guided cleavage of target mRNAs in tomato

*SlAP2* gene family members are miR172 targets in tomato [55, 58, 91] though few of these interactions are functionally characterized beyond the *APETALA2*/*ERF* gene, *SlAP2a* [42]. Because most plant miRNAs are complementarity to their target genes, miRNA-mediated cleavage is likely the predominant mechanism of gene regulation [18, 20, 92, 93]. Interestingly, overexpression of miR172 in *Arabidopsis* down-regulated *AP2* and *AP2*-like (*TOE1*–*2*) by translation repression [48–50, 69] though substantial accumulation of target gene cleavage products was noted. In this case transcript levels were not reduced due to feed-back regulated induction via decreased AP2 protein.

Tomato *miR172* has five predicted target genes including *SlAP2a*. We conducted 5′-RACE PCR for these genes and successfully identified cleavage sites in four, including *SlAP2a*. All identified cleavage events occurred near the middle of the complementary region and were identical to *Arabidopsis* miR172 cleavage sites (Fig. 10). There are at least three mature tomato isoforms of miR172. Using the modified RACE PCR we validated three target genes regulated by *SlmiR172a* and observed dramatically reduced *SlAP2a* transcript levels in *SlmiR172a* OE lines (Supplementary Fig. S1A and additional file 1). These results are consistent with guided target gene cleavage as the predominant mechanism of *SlmiR172* action, and support miR172 guided target gene cleavage [54]. However, reduced *SlAP2*a mRNA in *SlmiR172a* OE lines contrasts with observations in *Arabidopsis* [39, 48–50, 60], suggesting that tomato miR172s regulate transcription largely by cleavage and may be less influenced by feedback regulation.

### miR172 could be regulated by other transcription factors controlled by other miRNAs

*SlmiRNA172a* overexpression affected fruit ripening via *SlAP2a*. When we investigated the expression of key genes (*MADS-RIN, CNR*, *HB1*, and *NOR*) we found that mRNA levels were three times higher in the *Cnr* mutant than WT from the onset of ripening (30 days after 1 cm fruit) but not changed in the *rin*, *nor* or *Nr* mutants (Fig. 11 and additional file 1). *SPL-CNR* encodes an SBP-box (SQUAMOSA promoter binding protein-like, SPL) transcription factor, and the *Cnr* epimutation suppressed *SlCNR* expression and inhibited ripening [36, 94, 95]. *SlCNR* is expressed in a fruit-specific manner and highest at the onset of ripening, tracking up-regulation of *SlmiR172* in *Cnr* and suggesting *SlmiR172* is negatively regulated by SPL-CNR during ripening and additional transcriptional inducers.

SPL7 is a member of the SPL family and binds the GTAC motif and regulates expression of miR398, miR397, miR408, and miR857, which are also induced under copper deficiency in *Arabidopsis* [96, 97]. Repression of *Arabidopsis* SPL3 regulated by miR156 causes transition from vegetative phase to floral induction. *SlSPL-CNR* is an ortholog of *AtSPL3* (*At2g33810*) and contains the miR156 binding site in the 3′-UTR, implying that *SlSPL-CNR* could be regulated by *SlmiR156* at ripening. The miR172 transcript is down-regulated by the dominant *Congrass1* (*Cg1*) mutation and by overexpression of two tandem miR156 genes in maize, suggesting a converse regulatory relationship [98, 99].

## Conclusions

Ripening of fleshy fruits like tomato is controlled by a complex regulatory network of multiple genes influenced by numerous internal and external cues. Previously, we reported the role of *SlAP2a* in the ripening process as a negative regulator of tomato fruit ripening modulating ethylene production and carotenoid pigmentation. We focused on identification of miRNAs targeted to *SlAP2* genes regulating flowering and fruit ripening. We show that miR172 can repress *SlAP2a* transcript accumulation and enhance ripening by modulating ethylene and carotenoid pigmentation. Demonstration of miR172a-guided cleavage of *SlAP2a* transcripts provides a molecular mechanism for this aspect of ripening regulation and candidate genetic targets for modifying quality and nutritional value of fleshy fruit crops.

## Methods

### Construction of miR172a OE vector and plant transformation

A 403-bp fragment of predicted pre-miR172 sequence (Supplementary data 1) was amplified from BAC clone *Mbo*I0036L22-T7 by PCR with specific primers

(Fw 5′-GCGCGCTCTAGAGTATATATATGTACTTGGATTTGTA-3′,

Rev. 5′-GCGCGCGAGCTCGAACCCCAGTATATACAAAACCCT-3′).

The fragment of *miR172a* precursor was gel-purified, cut by *Xba*I and *Sac*I, and cloned into the transformation binary vector pBI121 under the control of the CaMV 35S promoter. The transformed plasmid vector was introduced into *A. tumefaciens* strain LBA4404 by electroporation and transformed into WT tomato cv. AC, as described [42].

### Plant material and treatments

Transgenic and control plants were grown under standard greenhouse conditions at Cornell University (Ithaca, NY, USA). The greenhouse was equipped with heating, cooling, and supplemental lighting systems, and the plants were supplied with a slow-release fertilizer. We collected different types and stages of tissue: L (leaf), F (flower), D20 (20 days after 1 cm fruit), MG (mature green fruit), B (breaker fruit), B1 (1 day after breaker), B3 (3 days after breaker), B7 (7 days after breaker). On account that fruits of control and transgenic lines reached the breaker stage in different days after 1 cm, MG-stage control and transgenic fruits were collected at 33 and 28 days after reaching 1 cm, respectively. Time to ripening was measured by tagging 1-cm (7–8 days post-anthesis) fruits and the number of days from 1 cm to B-stage was recorded. At least 25 fruit of each genotype were used for this experiment. Flowering time was counted by two different measurements (i) the number of days from sowing to full blooming flower, and (ii) the number of leaves produced below the first inflorescence.

### RNA isolation and gel-blot analysis

Different developmental stages of tomato fruit were collected, immediately frozen in liquid nitrogen, and stored at − 80 °C. Total RNA was extracted from a pooled sample of five plants, 25 flowers and fruits for vegetative tissue and flower, as well as fruit samples at each developmental stage, respectively, as detailed in the literature [100]. For quantifying the total RNA, a NanoDrop ND-1000 spectrophotometer (Thermo Fisher Scientific, Waltham, MA, USA) was used.

For RNA blot analysis, 20 μg of total RNA was fractionated on 1% (w/v) agarose gel containing 7.5% (v/v) formaldehyde and blotted onto Hybond N membrane (Amersham Biosciences, Piscataway, NJ, USA) following the method [42]. The membranes were incubated at 80 °C for 2 h and then pre-hybridized for 3 h, as per the membrane supplier’s protocol, and hybridized at 65 °C to ^32^P–labeled random-primed DNA probes, synthesized as described previously [101]. Hybridizations were performed for at least 16 h, and the filters were washed in 2X SSC containing 0.1% SDS (sodium dodecyl sulfate), and then 1X SSC containing 0.1% SDS at 65 °C. Signal intensity was visualized by autoradiography using Kodak X-OMAT-AR film (Sigma–Aldrich, St. Louis, MO, USA) with two intensifying screens at − 80 °C.

For miRNA analysis, 50 μg of total RNA was loaded on 20% polyacrylamide/7 M urea denaturing gels and blotted onto Hybond N membrane (Amersham Biosciences). Oligonucleotide antisense to *miR172a* was end-labeled with γ-^32^P using T4 polynucleotide kinase (New England Biolabs, Osaka, Japan) and hybridized to miRNA blots at 42 °C in hybridization buffer, which contained 5X SSC, 0.5% SDS, 50 mM potassium phosphate buffer (pH 6.5), and 5X Denhardt’s solution [102]. The filters were washed twice in a mixture of 2X SSC and 0.1% SDS at 42 °C. Signal intensity was captured, as described above.

### Ethylene and carotenoid measurements

Twenty-five fruit were harvested from each line at different ripening stages and pooled them for measuring the ethylene level and carotenoid content using a Hewlett-Packard HP 5890 series gas chromatograph equipped with a flame ionization detector, as detailed previously [103–106]. Fruits were harvested 3 h prior to measure ethylene and stored at room temperature for reducing harvest stress. Whole fruits were sealed in 250 ml airtight jars and kept for 2 h at room temperate for measuring ethylene production, 1 ml of headspace gas was taken from the chamber and injected into gas chromatograph (Hewlett- Packard 5890 series II; Hewlett-Packard, http://www.hp.com) equipped with flame ionization detector and activated alumina column. Ten (10) ppm ethylene was used as standard (Airgas, Inc., http://www.airgas.com) for quantifying Ethylene concentrations, and normalized by fruit mass.

Carotenoid extraction and HPLC quantification were accomplished following the method described by [104–106]. Two hundred (200) mg frozen pericarp was homogenized with the mixture of 600 μl tetrahydrofuran/methanol and 12.5 mg of MgCO_3_.2H_2_O. The homogenate was filtered through spinning with a spin-X filter, then re-extracted by adding 550 μl of tetrahydrofuran. One hundred fifty (150) μl of 25% NaCl was added to partition the carotenoids into 325 μl of petroleum ether. The extract was dried by evaporation. Dry extract was suspended into methyl t-butyl ether: methanol (500: 475), and filtrated through a syringe filter (GE Osmonics). A C30 reverse-phase column (250 × 4.6 mm) was used in HPLC and the separations were performed by using a summit HPLC system along with a PDA-100 photodiode array detector (Dionex Corporation, CA, USA). The elution gradients were run for 5 min to 100% methanol, 20 min to 95% t-butyl ether, 5 min to 95% t-butyl ether, and finally 5 min for returning the system to 100% methanol. Before each run the column was equilibrated for 10 min with 100% methanol. Purified standards and/or pigment-specific absorbance spectra was used for identifying the specific pigments.

### Modified 5′ RNA ligase-mediated RACE for mapping of mRNA cleavage site

RNA ligase-mediated rapid amplification of cDNA ends (5′ RACE) was performed using Gene Racer Kit (Invitrogen, CA, USA) A with few modification [20]. Large-scale total RNA was extracted from different developmental stages of fruit and flower using a hot phenol method [107] and treated with RNAase-free DNAase (RNeasy Mini Kit, Qiagen Sciences, MD, USA). The total RNA concentration was quantified using ND-1000 v3.1.0 (NanoDrop Technologies, Wilmington, DE). Poly (A)^+^ mRNA was extracted by an Oligotex mRNA Midi Kit (Qiagen, CA, USA) from 500 μM of total RNA.

Ligation of RNA adapter, reverse transcription, and 5′-RACE PCR were carried out according to the manufacturer’s instruction (Gene Racer Kit, Invitrogen) with 100 nM poly(A)^+^ mRNA. Non-gene specific 5′-RACE products were generated by amplification with the Gene Racer 5′ Primer (5′-CGACTGGAGCACGAGGACACTGA-3′) and Gene Racer 3′ Primer (5′-GCTGTCAACGATACGCTACGTAACG-3′). Gene-specific 5′-RACE reactions were conducted with the Gene Racer 5′ Nested Primer and gene-specific primers as follows: PCR products were gel-purified, cloned into a plasmid vector using the TA TOPO PCR Cloning Kit (Invitrogen), and sequenced using the Illumina platform.

### Semi-quantitative RT-PCR analysis

For RT-PCR analysis, first-strand cDNA was synthesized from 3 μg of total RNA using superscript III reverse transcriptase (Invitrogen) and oligo-dT primer according to the manufacturer’s protocol. PCR was conducted using *SlAP2b*-specific primers (Supplementary Table S1) following 1 cycle at 94 °C for 3 min; 30 cycles at 94 °C for 1 min, 50 °C for 2 min, and 72 °C for 10 min; where first-strand cDNA was used as a template.

## Supplementary information

**Additional file 1 **: **Supplementary Table S1.** List of specific primers used in RT-PCR

**Additional file 2 **: **Supplementary Fig. S1.** RACE-PCR analysis of miR172 cleaved products. Analysis was performed on wild-type and *miR172* OE lines for (**A**) ripening stage fruit (B3) and (**B**) flowers. A fragment of the *EF1-α* gene was amplified in the same PCR reaction as an internal control. **Supplementary Fig. S2.** Expression level of the *SlAP2a* gene in wild-type and *miR172* OE lines. WT and miR172 OE lines #16 and #26 tissues (L, leaf; B, breaker; B3, 3 days after breaker) were analyzed. The same filter was stripped and rehybridized to tomato a18S rRNA probe as a loading control. **Supplementary Fig. S3.** miR172 transcript abundance in wild-type (WT) and ripening mutants (*rin*, *nor*, *Nr*, *Cnr*). Tomato tissues (L, leaf; F, flower; D10, 10 days after 1 cm fruit; D20, 20 days after 1 cm fruit; D30, 30 days after 1 cm fruit; D35, 35 days after 1 cm fruit), were harvested, extracted for total RNA and 50 μg was loaded for RNA gel-blot analysis using a 5′-end-labeled DNA oligonucleotide probe complementary to miR172. 5S rRNA was used as a loading control. **Supplementary Fig. S4.** Accumulation of miR172 in wild-type and *Cnr* mutant fruit. Tomato tissues (F, flower; D10, 10 days after 1 cm fruit; D20, 20 days after 1 cm fruit; D30, 30 days after 1 cm fruit; D35, 35 days after 1 cm fruit) were extracted for total RNA of which 20 μg/sample was separated for RNA gel-blot analysis using a 5′-end-labeled DNA oligonucleotide complementary to miR172 as probe. Tomato 5S rRNA was used as a loading control. RNA gel-blot analysis was repeated three times and relative expression was determined relative to transcript levels in flower.

**Additional file 3 **: **Supplementary data 1.** Sequences of *AP2* family genes and putative miR172 binding sites

**Additional file 4.** : Additional file 1. Gel images of Northern blot and RACE-PCR electrophoresis performed for different genes and RNAs which are used in this manuscript.

## Data Availability

The datasets used and/or analysed during the current study available from the corresponding author on reasonable request.

## Abbreviations

*ACO1*: *1-AMINOCYCLOPROPANE-1-CARBOXYLIC ACID OXIDASE*;
*ACS2*: *1-AMINOCYCLOPROPANE 1-CARBOXYLIC ACID SYNTHASE*
*AP2*: *APETALA2*
B: Breaker fruit
B1: 1 day after breaker
B3: 3 days after breaker
B7: 7 days after breaker
BAC: Bacterial Artificial Chromosome
*Cnr*: *Colorless non-ripening*
*CO*: *CONSTANS*
*CRY*: *Cryptochromes*
*CYC-B*: *Lycopene-β-cyclase*
D20: 20 days after 1 cm fruit
DCL: Dicer-like
*DXS1*: *1-deoxy-D-xylulose 5-phosphate synthase*
F: Flower
*FT*: *FLOWERING LOCUS T*
*gf*: *green flesh*
*GI*: *GIGANTEA*
*gl15*: *glossy15*
*Gr*: *Green-ripe*
*HB1*: *HD-ZIP HOMEOBOX PROTEIN-1*
HPLC: High performance liquid chromatography
L: Leaf
*RIN*: *RIPENING INHIBITOR*
MG: Mature green fruit
miRNA: MicroRNA
ND: NanoDrop
*NOR*: *NON-RIPENING*
*Nr*: *Never-ripe*
OE: Overexpression
PDA: Potato Dextrose Agar
*PDS*: *Phytoene desaturase*
*PHY*s: *Phytochromes*
*PI*: *PISTILLATA*
pri-miRNA: primary transcript RNA
*PSY1*: *Phytoene synthase 1*
RISC: RNA-induced silencing complex
RNA: Ribo Nucleic Acid
*SFT*: *SINGLE FLOWER TRUSS*
*TOE1*: *TARGET OF EAT1*
*TOE2*: *TARGET OF EAT2*
WT: Wild
*ZDS*: *ζ-carotene desaturase*
