# Supplementary material for: Ectopic expression of miRNA172 in tomato (Solanum lycopersicum) reveals novel function in fruit development through regulation of an AP2 transcription factor
Source: BMC Plant Biol. 2020 Jun 19;20:283. doi: 10.1186/s12870-020-02489-y (PMC7304166; doi:10.1186/s12870-020-02489-y)
Supplement: Supplementary file 1 — Additional file 1 : Supplementary Table S1. List of specific primers used in RT-PCR [file 12870_2020_2489_MOESM1_ESM.docx]

**Supplementary Table S1.** List of specific primers used in RT-PCR

| **Annotation** | **TC and**  **SGN no.** | **Gene Bank**  **accession** | **Forward (5′…………. 3′)** | **Reverse (5′…………. 3′)** | **Amplicon size (bp)** |
| --- | --- | --- | --- | --- | --- |
| *SlAP2b* | TC 180296 | BI208808 | CATCAAGTGTAACGGGAAGGATG | GGAAACCATTTTCTGAGGACCA | 653 |
| *SlAP2c* | SGN 314858 | BI935838 | TGATATGGAGTATGGGCGAAGAA | GTGGATGGTATAAATTGGCAGCTT | 625 |
| *Target4* | SGN 325104 | BG627220 | GACAGCGAAGTAGAAGCTGCAAG | TAGGCGGGGATTAATCTGGTAAA | 591 |
| *Target5* | SGN 325757 | BP893968 | CTCACTTGTGGAATGGAGCATATT | GGTAGTTGTGGCCTGATCTGGTA | 316 |
| *SlEF1-α* |  | CP023762.1 | TCAGGTAAGGAACTTGAGAAGGAGCCT | AGTTCACTTCCCCTTCTTCTGGGCAG | 110 |
