# Supplementary material for: Ectopic expression of miRNA172 in tomato (Solanum lycopersicum) reveals novel function in fruit development through regulation of an AP2 transcription factor
Source: BMC Plant Biol. 2020 Jun 19;20:283. doi: 10.1186/s12870-020-02489-y (PMC7304166; doi:10.1186/s12870-020-02489-y)
Supplement: Supplementary file 2 — Additional file 2 : Supplementary Fig. S1. RACE-PCR analysis of miR172 cleaved products. Analysis was performed on wild-type and miR172 OE lines for (A) ripening stage fruit (B3) and (B) flowers. A fragment of the EF1-α gene was amplified in the same PCR reaction as an internal control. Supplementary Fig. S2. Expression level of the SlAP2a gene in wild-type and miR172 OE lines. WT and miR172 OE lines #16 and #26 tissues (L, leaf; B, breaker; B3, 3 days after breaker) were analyzed. The same filter was stripped and rehybridized to tomato a18S rRNA probe as a loading control. Supplementary Fig. S3. miR172 transcript abundance in wild-type (WT) and ripening mutants (rin, nor, Nr, Cnr). Tomato tissues (L, leaf; F, flower; D10, 10 days after 1 cm fruit; D20, 20 days after 1 cm fruit; D30, 30 days after 1 cm fruit; D35, 35 days after 1 cm fruit), were harvested, extracted for total RNA and 50 μg was loaded for RNA gel-blot analysis using a 5′-end-labeled DNA oligonucleotide probe complementary to miR172. 5S rRNA was used as a loading control. Supplementary Fig. S4. Accumulation of miR172 in wild-type and Cnr mutant fruit. Tomato tissues (F, flower; D10, 10 days after 1 cm fruit; D20, 20 days after 1 cm fruit; D30, 30 days after 1 cm fruit; D35, 35 days after 1 cm fruit) were extracted for total RNA of which 20 μg/sample was separated for RNA gel-blot analysis using a 5′-end-labeled DNA oligonucleotide complementary to miR172 as probe. Tomato 5S rRNA was used as a loading control. RNA gel-blot analysis was repeated three times and relative expression was determined relative to transcript levels in flower. [file 12870_2020_2489_MOESM2_ESM.ppt]

## Slide 1
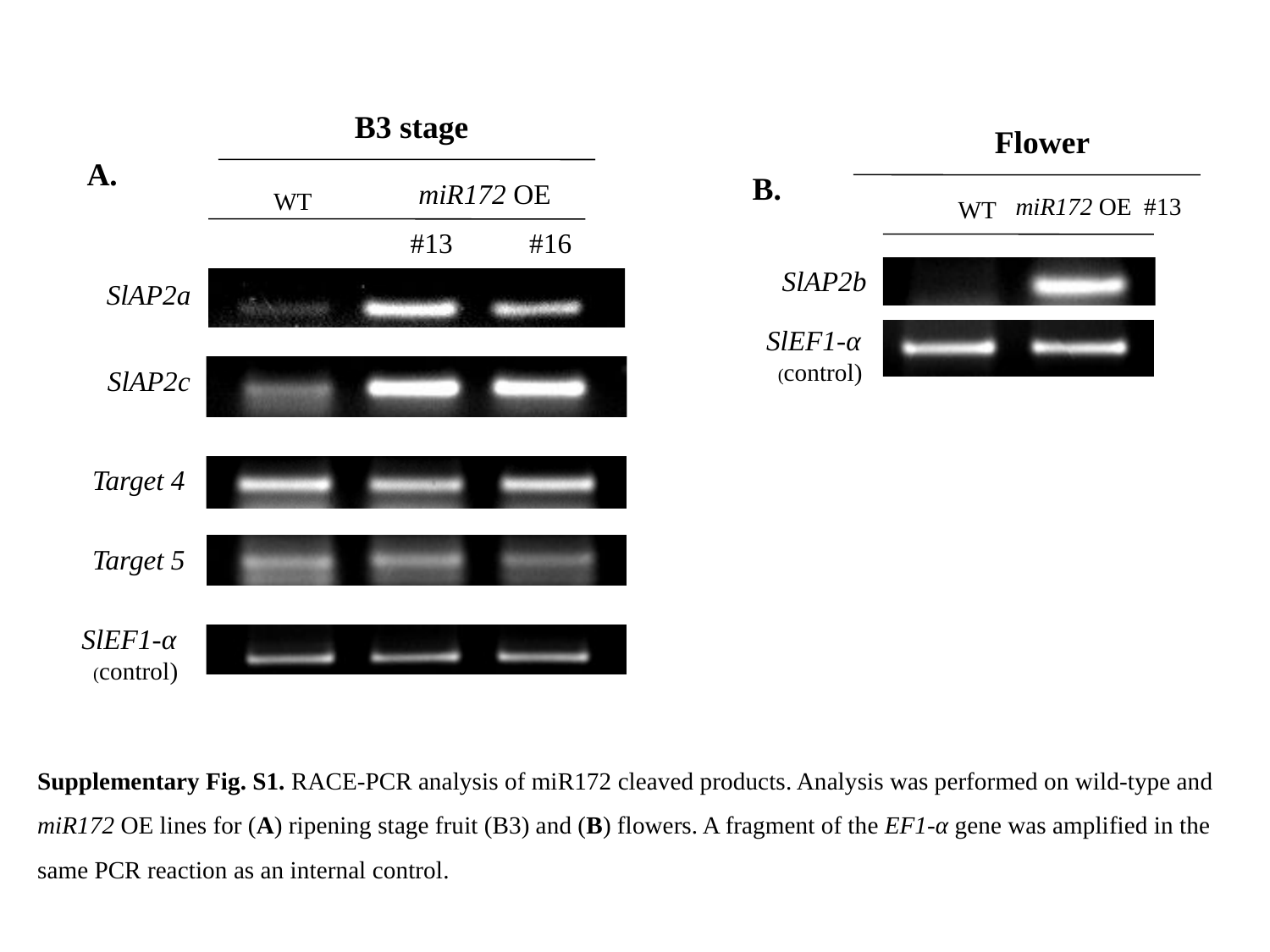

B3 stage
 miR172 OE
WT
#13
#16
Flower
miR172 OE #13
WT
A.
B.
SlAP2b
SlAP2a
SlEF1-α
 (control)
SlAP2c
Target 4
Target 5
SlEF1-α
 (control)
Supplementary Fig. S1. RACE-PCR analysis of miR172 cleaved products. Analysis was performed on wild-type and miR172 OE lines for (A) ripening stage fruit (B3) and (B) flowers. A fragment of the EF1-α gene was amplified in the same PCR reaction as an internal control.

## Slide 2
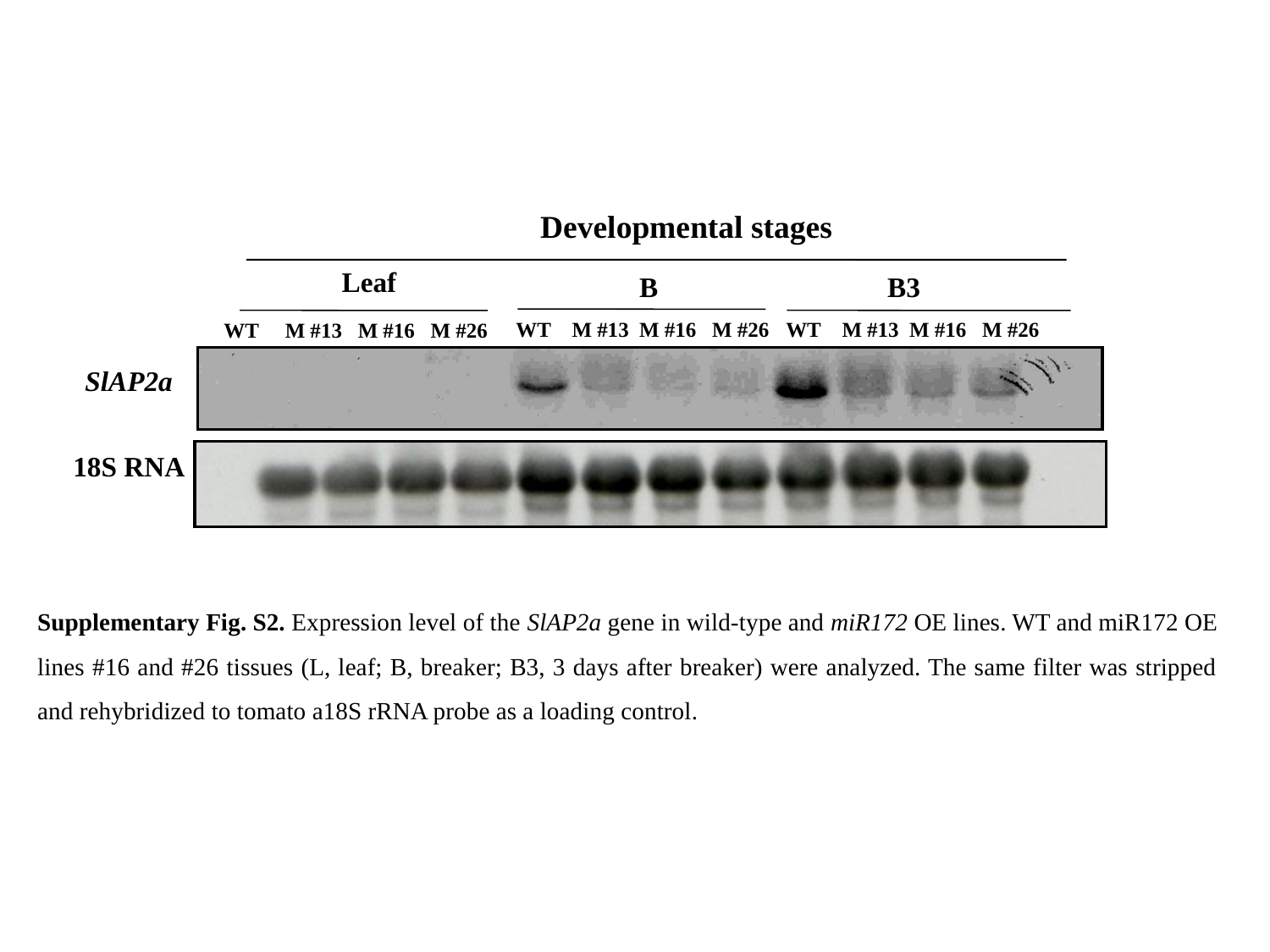

Developmental stages
Leaf
B
B3
WT M #13 M #16 M #26
WT M #13 M #16 M #26
SlAP2a
18S RNA
WT M #13 M #16 M #26
Supplementary Fig. S2. Expression level of the SlAP2a gene in wild-type and miR172 OE lines. WT and miR172 OE lines #16 and #26 tissues (L, leaf; B, breaker; B3, 3 days after breaker) were analyzed. The same filter was stripped and rehybridized to tomato a18S rRNA probe as a loading control.

## Slide 3
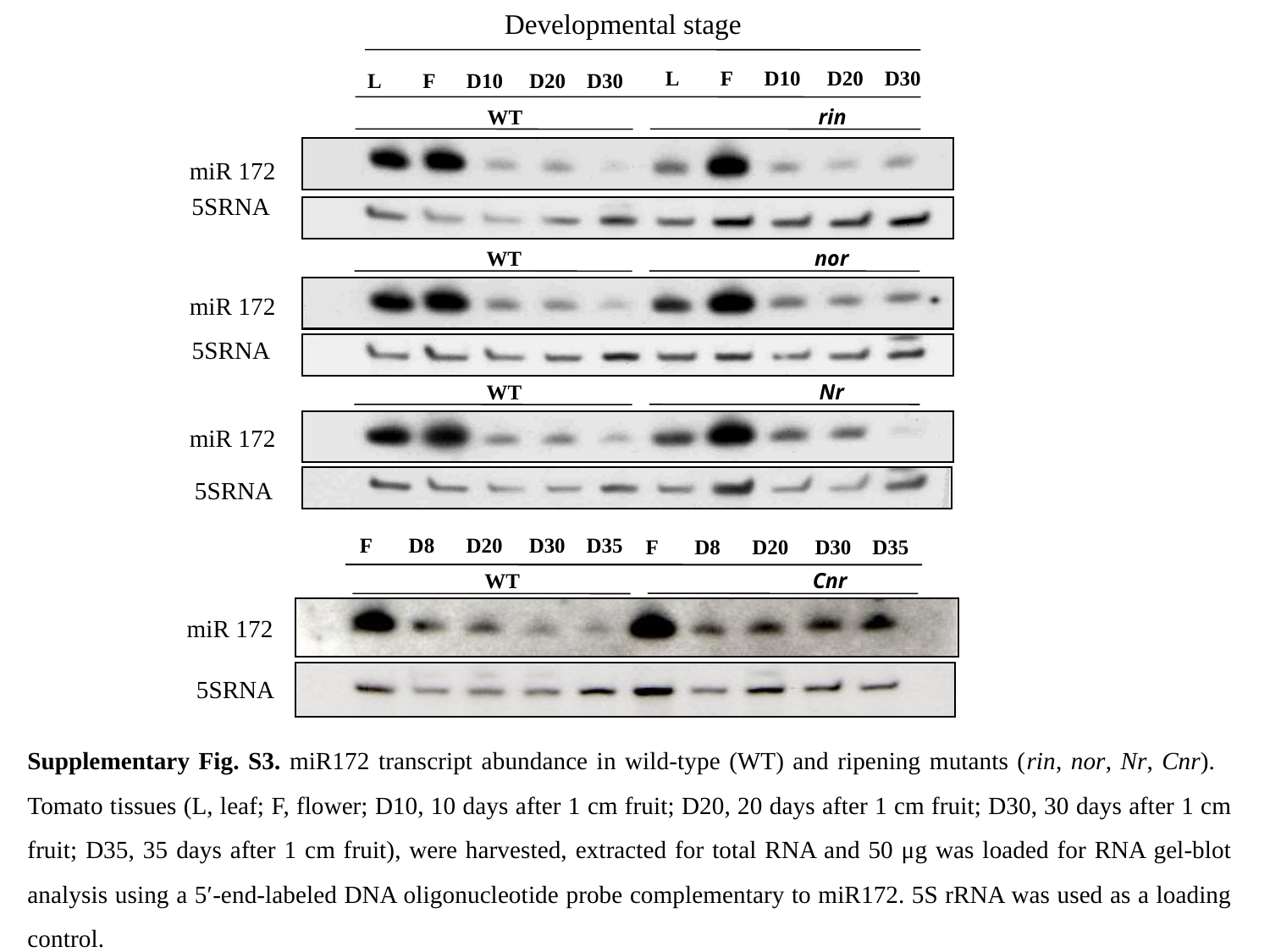

Developmental stage
L F D10 D20 D30
L F D10 D20 D30
WT
rin
miR 172
5SRNA
WT
nor
miR 172
5SRNA
WT
Nr
miR 172
5SRNA
F D8 D20 D30 D35
F D8 D20 D30 D35
WT
Cnr
miR 172
5SRNA
Supplementary Fig. S3. miR172 transcript abundance in wild-type (WT) and ripening mutants (rin, nor, Nr, Cnr). Tomato tissues (L, leaf; F, flower; D10, 10 days after 1 cm fruit; D20, 20 days after 1 cm fruit; D30, 30 days after 1 cm fruit; D35, 35 days after 1 cm fruit), were harvested, extracted for total RNA and 50 μg was loaded for RNA gel-blot analysis using a 5′-end-labeled DNA oligonucleotide probe complementary to miR172. 5S rRNA was used as a loading control.

## Slide 4
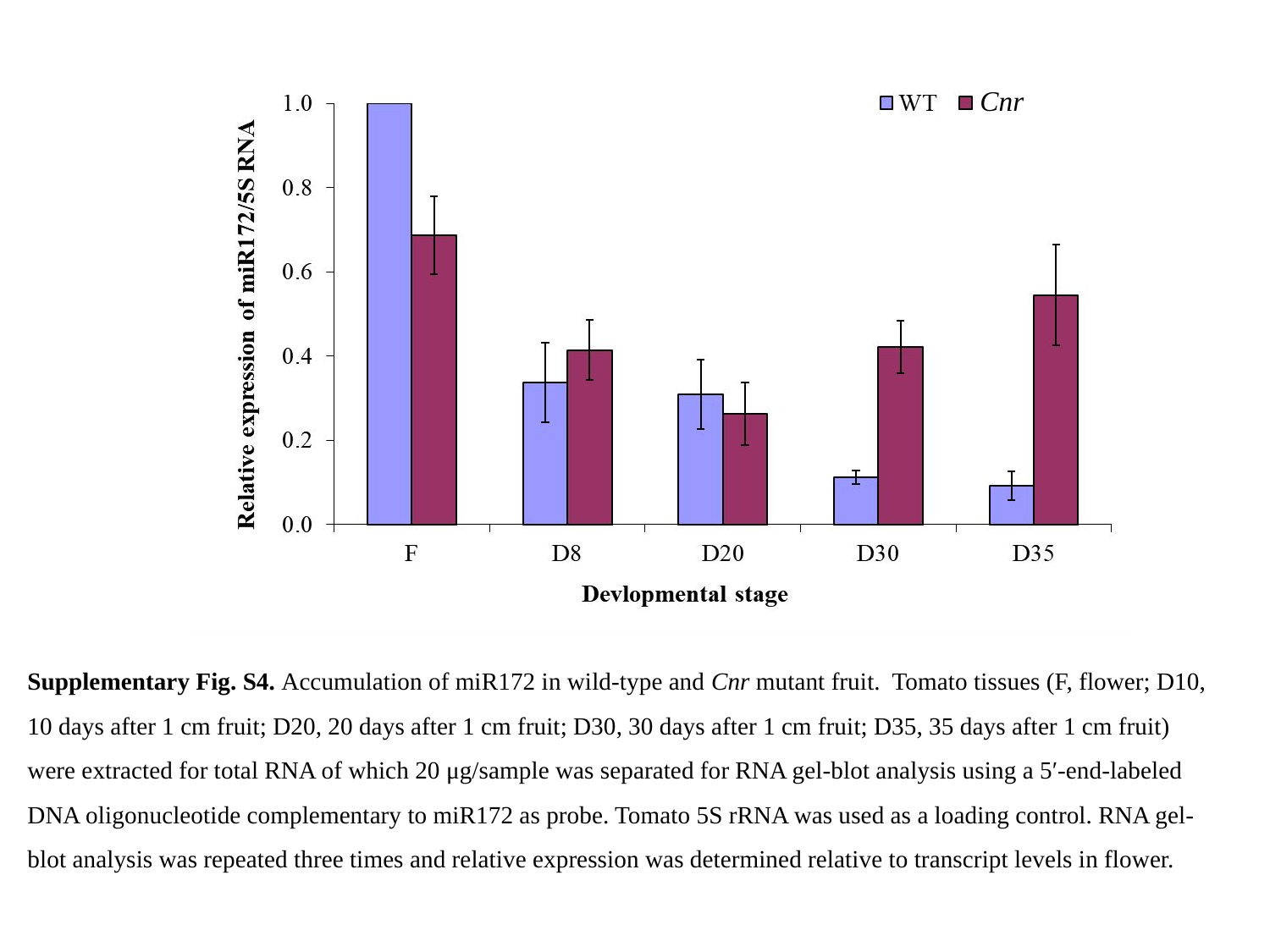

Cnr
Supplementary Fig. S4. Accumulation of miR172 in wild-type and Cnr mutant fruit. Tomato tissues (F, flower; D10, 10 days after 1 cm fruit; D20, 20 days after 1 cm fruit; D30, 30 days after 1 cm fruit; D35, 35 days after 1 cm fruit) were extracted for total RNA of which 20 μg/sample was separated for RNA gel-blot analysis using a 5′-end-labeled DNA oligonucleotide complementary to miR172 as probe. Tomato 5S rRNA was used as a loading control. RNA gel-blot analysis was repeated three times and relative expression was determined relative to transcript levels in flower.
