# Supplementary material for: Ectopic expression of miRNA172 in tomato (Solanum lycopersicum) reveals novel function in fruit development through regulation of an AP2 transcription factor
Source: BMC Plant Biol. 2020 Jun 19;20:283. doi: 10.1186/s12870-020-02489-y (PMC7304166; doi:10.1186/s12870-020-02489-y)
Supplement: Supplementary file 3 — Additional file 3 : Supplementary data 1. Sequences of AP2 family genes and putative miR172 binding sites [file 12870_2020_2489_MOESM3_ESM.doc]

**Supplementary data 1.** Sequences of *AP2* family genes and putative miR172 binding sites

1. **Sequences of the *AP2* family genes harbor the miR172 binding sequence**

***AP2a* (SGN 314860 partial)**

ATAAGCTGCTTTAGGCTTATTCTTCCGAAACTAGCCTCGTCTATATCCATCATCTCTCTCTTAATTTTTATTTTTTTTCTTTCCCCAACAACTTCAACTTCTCTATAAGTCATAACTTCCATCTTTCTTTCAATTTACAACAAAAAAATAAATAAATCATTCTTTTCTTCATTTCATACATCAATATAGGTCACTTGCAACCAAATATCGAAAAATACACTCATTTTAATTCTATCGACAAAAGAAATGTGGAATTTAAATGATTCCCCTGATCAGACAATGGAGTATGAATCCGATGAAGGTATAACGGTTAGATCGGAATCAAATTCAATTTCATCCGCTCTACTTGTTGTAGAAGACGGTAATTCATCAGAAGAAGACGGTGAAAAAGGTAAAAAAAAGAAAAGTAATAATACACCAGGTAAAATATTCGGATTTTCGATTAAAGACCACAATTTGGAATCGCCGGTAGTTACCCGGCAATTTTTTCCGGTAGATAACGAGTCTACCAATTTTCCAAGGGCACAATGGGCTGGAATTAAATTTTGTGAATCGGAACCGCCACTTGTCAATGGATTAGTGGGGAACAAAATTGACGTGTTGCAACAACAGCCAATTAAAAAAAGCCGTCGTGGACCACGTTCAAGAAGTTCACAGTACCGTGGTGTTACCTTTTACCGGAGGACTGGCCGGTGGGAATCTCATATATGGGATTGTGGGAAACAAGTGTATTTAGGTGGATTTGATACAGCACATGCAGCAGCTCGTGCATATGATAGGGCAGCAATCAAATTCCGGGGAGCGGAGGCGGACATAAACTTTACCTCGAAAGATTATGAAGATGACTTAAAACAGATGAGCAATTTAACCAAAGAAGAATTTGTGCATGTACTAAGGAGACAAAGTACTGGTTTTCCAAGAGGAAGTTCCAAGTATAGAGGAGTCACTTTGCATAAATGTGGTAGATGGGAAGCTAGAATGGGACAATTCTTAGGCAAAAAGTATGTTTATTTAGGCCTATTTGATACTGAAGTTGAAGCTGCCAGAGCTTATGATAAAGCTGCTATCAAGTGTAATGGAAAAGATGCAGTTACCAACTTTGATCGTAGCATTTATGAAAATGAACTTAACTCAACCGAATGTACTGATAATGCAACGGACCACAATCTTGACTTAAGCTTAGGAGGTTCAAGCCAAGAAATGGGGGACAATAGGGGTCAAAATTCTTCATCTAATTTGCAATTGGATGGCCATTGGGGCCACCAAGGGTCATCAAGGCATAATAATAAGGTTCAGACTCCGAGCAGCAACAATGTAGGCCAAATTGGTGGAACAAGTAATTTTCAAAATAATTATGAGATAATTGCAACTGCTGCAGCATCATCAGGATTCCCCCAGCAGATAATAAGGACTCAAAATAATGGCTTCCATCATTATTTTATGAGACCTTGACCATTCATTAATTTCTTCATGTTCTTTTATTAATCTTTGTAAAGAGTAAAATCAATTAATGTTGAGATTATAATTAATATTCTCAAAACATAAACAAACAGATTATATTGGTTAGTATATATATGTTGGGAGGAAATTCCAACTCTTTGGAAAAGTTAAGTGTAACCTTTTTTTTTTTTGTTAAATTATTATTATTATTGGGACAATGTAATTTSAATTTTTTGGAT

***AP2b* (SGN 314858 full)**

AAAGAAAAGAATAGAGATAGAGAGAGAAAATAGAGGAGAATATTTAAAAGTCTAGGAAACTCAAAACAAGTCCTTTTGTAGCAATAGCCAAAAAATTAAATTGTTTTCTGTTTTTATAATTTGGATTGGGTATTTTTTGTAGAGAGAAAAATGTGGGATCTAAATGAATCAGAAGAAGGTTGTTCTTCACCGATAGAGTTTGAAGGCGATGATGAGAAAGGTAAACGGGTCGGATCCGTTTCGAATTCAAGTTCATCAGCTGTAGCTGTTGATGATATATCTGAAGAGGAATTAGATGGAGAAAGAGGGAAGAAAAAGAGAGGTAAAATTTTCGGCTTCTCTATGGTGGGTCTGGGTAACGGCGATGAAGAACAGCCGGTGACCCGACAGTTTTTTCCGGTTGATGAGTCGGAAATGGGTGGTGTTGCTGCTGAAAATGGATGCCCGAATTTTCCCAGAGCTCACTGGGTTGGAGTTAAATTTTACCAAACGGAGACACTTGGCAACACGGGATTGGCCAGGCCTGTAGATATGGTTCAGCAGCAACAGCAGCCTATTAAGAAGAGCCGCCGTGGACCAAGGTCTAGAAGCTCACAGTACCGTGGGGTTACCTTCTACCGGAGAACTGGCCGGTGGGAATCTCATATATGGGATTGTGGAAAACAAGTTTATCTAGGTGGATTTGATACTGCTCATGCAGCAGCTCGGGCATACGATAGGGCAGCCATAAAATTCCGGGGAGTAGAAGCCGACATAAACTTTAGCTTAGAAGATTATGAGTCCGATTTAAAACAGATGACAAGTTTAACAAAGGAAGAATTTGTGCATGTGCTTCGAAGACAAAGTACTGGTTTTCCGAGGGGAAGCTCCAAGTATAGAGGGGTGACTTTGCACAAATGTGGTAGATGGGAAGCTAGAATGGGACAGTTCTTAGGCAAAAAGTACGTTTATTTGGGACTCTTTGATACTGAGATTGAAGCTGCCAGAGCGTATGATAAAGCTGCCATCAAGTGTAACGGGAAGGATGCAGTTACAAATTTTGATCCAAGCATTTATGAGAATGAACTAAACTCAATTGAATCTACCGATAACGTGGCTGATCATAATCTGGATTTAAGTTTGGGTGGTTCAAGCTCAAAGCAAGGAAGTCAAGAATTGGGGGATAATAGAGGTCAAAATTCTTCATCAATAATGCAATTGGATATTGATTGGCAGCGCCATGGTTTAAGGCCTGAGAAGCAATCTGCCTTAATTGATGCTCGAAGAAGAGAAAACAGGTACAATGAAACAGAAACGTTGCAACTTTTAAGCCAGACGCACTTGCATTCTCCAGCCTCCTTGAAGCATAACAATAATAATAATAGTCAAGTGCAACGGTTTGGCCAATTTATGAGACCTGGTGATCAATCCCATATGATTCAAATGTTTCCACAGCAATTTGGCTCATCAAATTATCAAATTCAATTTCCAAGTGGCAGCAATGGTGGAAGAATTGGAGCTACAAATGTAAGAGATCTATCGCTTGCGGCGACGAGTAATGGTTCTTCACAATGGCAATCCAATTTTCCTCCTCAGATATTTGTAGCTGCTGCAGCATCATCAGGATTCCCTCAGCAGATAGTAAGACCTCAGAATTGGTCCTCAGAAAATGGTTTCCATCATTCACTCATGAGACCTTCTTGACTCATTTAATAACTTGCCATATTTCTATTTGTGTTTTTTTTAAGATAATGTGTAGATTAAAGGGTTGATATTCTTAATACTCCATATATATTACCTAAGGCTCTTGGTTTGAAATCAAAAAAAAAAAAAAA

***AP2c* (SGN 314858)**

TCAGAAGAAGGTAAAGGTGTTGGATCCATTTCAAATTCAAGTTCATCAGCTGTTGAGGAATTATACGGTTCAGAGGAAGAAGATGAATTTCTCGAACAAAATGGACTAAAAGGCAAGAAAAAGAAAAATATTCCTAGTAAAATATTTGGCTTCTCTATGATTGCTCCACCTAATAATAACAATCACAACGACGATAACTTGTCGTCGGAGAGTGAACCGCCGGTTACCCGGCAGTTTTTTCCGGTTGATGAGTCGGAAATCGGAAGTGGCAATTTTAATGATAGATCTTGTAGATTTCCAAGGGCCCATTGGGCTGGAGTGAAATTCTACCAACCGGAGCCATCTGCTAATTCGCCAGCACTATTGGGGAAGGGTAGTGAGTTGTCGCAACAAGTGCAGCCAATGAAAAAAAGCCGGCGTGGTCCAAGGTCTAGAAGCTCACAGTACCGCGGTGTTACTTTTTATCGGAGAACTGGCCGCTGGGAATCACATATATGGGATTGTGGAAAGCAAGTTTATCTAGGTGGATTTGATACTGCACATGCTGCTGCTCGTGCGTACGATCGGGCAGCAATAAAATTTCGTGGAATGGAGGCGGACATAAACTTTAACCTCGAAGATTATGAGGAAGATTTAAAACAGATGAAGAATTTAACGAAGGAAGAATTTGTTCATGTACTGAGGAGGCAAAGTACTGGTTTTCCGAGGGGAAGTTCTAAGTATAGAGGGGTGACATTGCACAAATGTGGTAGATGGGAAGCTAGAATGGGTCAATTGTTGGGCAAAAAGTACGTTTATTTGGGACTCTTTGATACTGAAAATGAAGCTGCCAGGGCTTATGATAAAGCTGCCATCAAATGTAATGGCAAGGATGCAGTCACTAACTTCGATCCTTGCATTTATGAAAATGAACTTAATTCATCTGAATGTAGTAATAAAGCAGCAGATCACAGCCTTGACTTAAGCTTAGGTAGTTCGAGCTCAAAGCAAAACAGCCGTGAAATGGAGGATACTAATAAGAATCAAAATTATCCTTCTATCCAATTTGATGTTGATTGGCGACACCAAGGGTCGAAACCCAAGCAACCTAGTCCACTTGATATGGAGTATGGGCGAAGAAGAAATGTGTACAATGAGACGGAAACGTTGCAACTCTTTAGCCAAACGCACCTACATTCTCCGGGATCCTTCAAGACAAATAGCAATGAAATGCAAAGGTTTGGCCAATATATGAGAGCTGGTGACTCCCAAATGATTCCACCACAATTCACCTCCTCAAATTATCAAGTTCAGTTTCCAAGCAGCAGCAATAGAAGAGATACTCAACAATGGCAAAGCAATACTGTTCCTCCTCCTCACCTATTTGCAACTGCTGCAGCATCATCAGGATTCCCTCAGCAGATATTAAGACATCAAAACTGGCCTCACAAAAGTGGCTTTAACTACTCTCTCACCAGACCATCCAATTAATTTTCTATCGAGACAGTCGATCGATGCATGGAATATTTTGCGTCTAAGAGAGTTAGCGAACCGCACGCACCTGAAGGAGGGTTGTGATGTAAACTATAATGTAATATAATATTTACAAGGGTTAATCTTGATGGTAAAAAAAAGCTTTTGAGAGATTCCAACTCTATGGATATAGTTGGTACAAAGCTAACTGAAGAAGAGATATTGAACTAGATTTCTTACTCATGTAAGCTGCCAATTTATACCATCCACAAGTACAGCTACTAAATAAATGTTTTTAATTTTTTGGTGGTCTATCTTGTGACTTATATGTTGTTATTTACCAATACATAATTCTCACTTTATTCCA

***Target4* (SGN 325104)**

TGCAATTAAGTTTAGGGGTGTTGATGCTGATATCAACTTTAGCTTAAGTGATTACGAGGAGGATATGCATCAGATGAAAAACCTTGGTAAAGAAGAATTTGTGCACTTGCTGCGACGCCATAGCACTGGTTTCTCAAGAGGGAGCTCCAAATTCAGAGGAGTGACGCTACATAAATGTGGCAGATGGGAAGCTCGGATGGGACAGTTCCTCGGGAAAAAGTATATATATCTTGGGCTGTTCGACAGCGAAGTAGAAGCTGCAAGGGCCTACGATAAGGCGGCAATTAAAACTAGCGGAAGGGAAGCTGTTACCAACTTTGAGCCAAGTAGCTATGAAGGGGAAACAATGTCTTTACCACAGAGTGAAGGTAGCCAACATGATCTTGATCTGAACTTGGGGATATCGACCACTTCTTCAAAGGAAAATGACAGGTTGGGAGGTTCTCGCTATCATCCTTACGATATGCAAGACGCAACAAAACCTAAGATGGATAAACCTGGTTCAGTAATAGTTGGAAGTTCACATCTCAAGGGACTACCAATGTCGTCCCAACAAGCTCAATTGTGGACTGGAATCTATTCTAATTTCTCTTCCAGCTATGAGGGAAGAGCATATGACAAGAGAAAGGACACAGGTTCATCACAAGGACCTCCAAATTGGGCACTGCAAATGCCTAGTCAGGTTGATACAAACAGCCCATTGACAATGTTCTGCACGGCAGCATCATCAGGATTCTTCATTCCATCTACTACTTCTATCACTTCATCAACATCTGCATTAGCAACTTCAACAAATGCCTCGCAGTGCTTTTACCAGATTAATCCCCGCCTACCACTTCCATAACATGTACTTATCCGTAACTAGTAATATAGGTATAAACATAGTCATTGATTTCTTTTGTATGAATAACATTTTTGTGGATTGGATAATTTTTAGAACTGCTTCAGGATCTGATAGGTAAAGATTTGAACTATACTTACAGATTGATATAGTATTTGTACTTATCCTTGCTTAAAAAAAAAAAAAAAAAAA

***Target5* (SGN 325757)**

CTCACTTGTGGAATGGAGCATATTCCAATTTAATTCCCAACTATGAGGAAAGAGTATCCGGGAAGAGAATAGACGTAGGTACCTCCCACGGACACCCAAACTGGGCAATGCAAATGCATAGTCAGGTCGGAACAACGCCATTGTCAATGTTCTCTGCTGCAGCATCATCAGGATTCTCAACTCCGGCTACCACTGCTTCGGCCTCTCAAATGTCTGGTCCCAACAACCCCAACACTCTCAATCTTTCGTTTGCTTCATATTCGTCACCATCAACGAATACAGCTCAATACTATTACCAGATCAGGCCACAACTACCACCTCCATAACATGTCTTACTATAAATACATTTACATAGTTAACTCAAAAACTGATTCCATTTGTAAGAATTGTTTGTTATCAATTCATTTACATGTTGATTTTAAGTTACAACTTGTAAGAATTGGAATGTGAAGATTCAAATTTCCATCACATTTTGAGCTTTTCCTTGCTTATTGTTGTGATTTAGAAGCAAAAGAAGCTCAGCTATAAAAAAAAAAAAAAAAAAAACTCGAGGGGGGCCCG

**B) Sequences of *AP2* genes specific to miR172 binding site**

***AP2a* (SGN 314860 partial)**

CAAATTGGTGGAACAAGTAATTTTCAAAATAATTATGAGATAATTGCAACTGCTGCAGCATCATCAGGATTCCCCCAGCAGATAATAAGGACTCAAAATAATGGCTTCCATCATTATTTTATGAGACCTTGACCATTCATTAATTTCTTCATGTTCTTTT

***AP2b* (SGN 314858 full)**

AATTTTCCTCCTCAGATATTTGTAGCTGCTGCAGCATCATCAGGATTCCCTCAGCAGATAGTAAGACCTCAGAATTGGTCCTCAGAAAATGGTTTCCATC

***AP2c* (SGN 314858)**

GGCAAAGCAATACTGTTCCTCCTCCTCACCTATTTGCAACTGCTGCAGCATCATCAGGATTCCCTCAGCAGATATTAAGACATCAAAACTGGCCTCACAAAAGTGGCTTTAACT

***Target4* (SGN 325104)**

TCACAAGGACCTCCAAATTGGGCACTGCAAATGCCTAGTCAGGTTGATACAAACAGCCCATTGACAATGTTCTGCACGGCAGCATCATCAGGATTCTTCATTCCATCTACTACTTCTATCACTTCATCAACATCTGCATTAGCAACTTCAACAAATGCCTCGCAGTGCTTTTACCAGATTAATCCCCGCCTACCACTTCCATAACATGTACTTATCCGTAACTAGTAATATAGGTATAAACATAGTCATTGATTTCTTTTGTATGAATAACATTTTTGTG

***Target5* (SGN 325757)**

ACCTCCCACGGACACCCAAACTGGGCAATGCAAATGCATAGTCAGGTCGGAACAACGCCATTGTCAATGTTCTCTGCTGCAGCATCATCAGGATTCTCAACTCCGGCTACCACTGCTTCGGCCTCTCAAATGTCTGGTCCCAACAACCCCAACACTCTCAATCTTTCGTTTGCTTCATATTCGTCACCATCAACGAATACAGCTCAATACTATTACCAGATCAGGCCACAACTACCACCT

**C) Potential targets genes of AP2 family harbor miR172 with specific binding site in tomato**

| **Annotation** | **SGN &**  **TC No** | **Gene Bank** | **Predicted target protein** | **miR172 binding sequence** |
| --- | --- | --- | --- | --- |
| *SlAP2a* | SGN 314860  TC172597 | BM410833  BM408872  BM413247  BE436427  BE432648 | PPHAP2A protein [*Petunia × hybrida*] | CTGCAGCATCATCAGGATTCC  ATGCAGCATCATCAAGATTCT |
| *SlAP2b* | SGN 314858  TC 180296 | BI208808 | PHAP2A protein [*Petunia × hybrida*] | CTGCAGCATCATCAGGATTCC  ATGCAGCATCATCAAGATTCT |
| *SlAP2c* | SGN 314858 | AI486618  BI935838  AI774952 | PHAP2A protein [*Petunia × hybrida*] | CTGCAGCATCATCAGGATTCC  ATGCAGCATCATCAAGATTCT |
| *Target 4* | SGN 325104 | BG627220 | Pathogenesis-related transcriptional factor and *ERF* (in *Medicago*)  RAP2.7/TOE1 (TARGET OF EAT1 1); DNA binding / transcription factor (in *Arabidopsis*) | CGGCAGCATCATCAGGATTTT  ATGCAGCATCATCAAGATTCT |
| *Target 5* | SGN 325757 | BP893968 | Pleckstrin homology (PH) domain-containing protein-related (in *Arabidopsis*) | TTGCAGCATCATCAGGATTCT  ATGCAGCATCATCAAGATTCT |
