# Supplementary material for: Ectopic expression of miRNA172 in tomato (Solanum lycopersicum) reveals novel function in fruit development through regulation of an AP2 transcription factor
Source: BMC Plant Biol. 2020 Jun 19;20:283. doi: 10.1186/s12870-020-02489-y (PMC7304166; doi:10.1186/s12870-020-02489-y)
Supplement: Supplementary file 4 — Additional file 4. : Additional file 1. Gel images of Northern blot and RACE-PCR electrophoresis performed for different genes and RNAs which are used in this manuscript. [file 12870_2020_2489_MOESM4_ESM.pptx]

## Slide 1
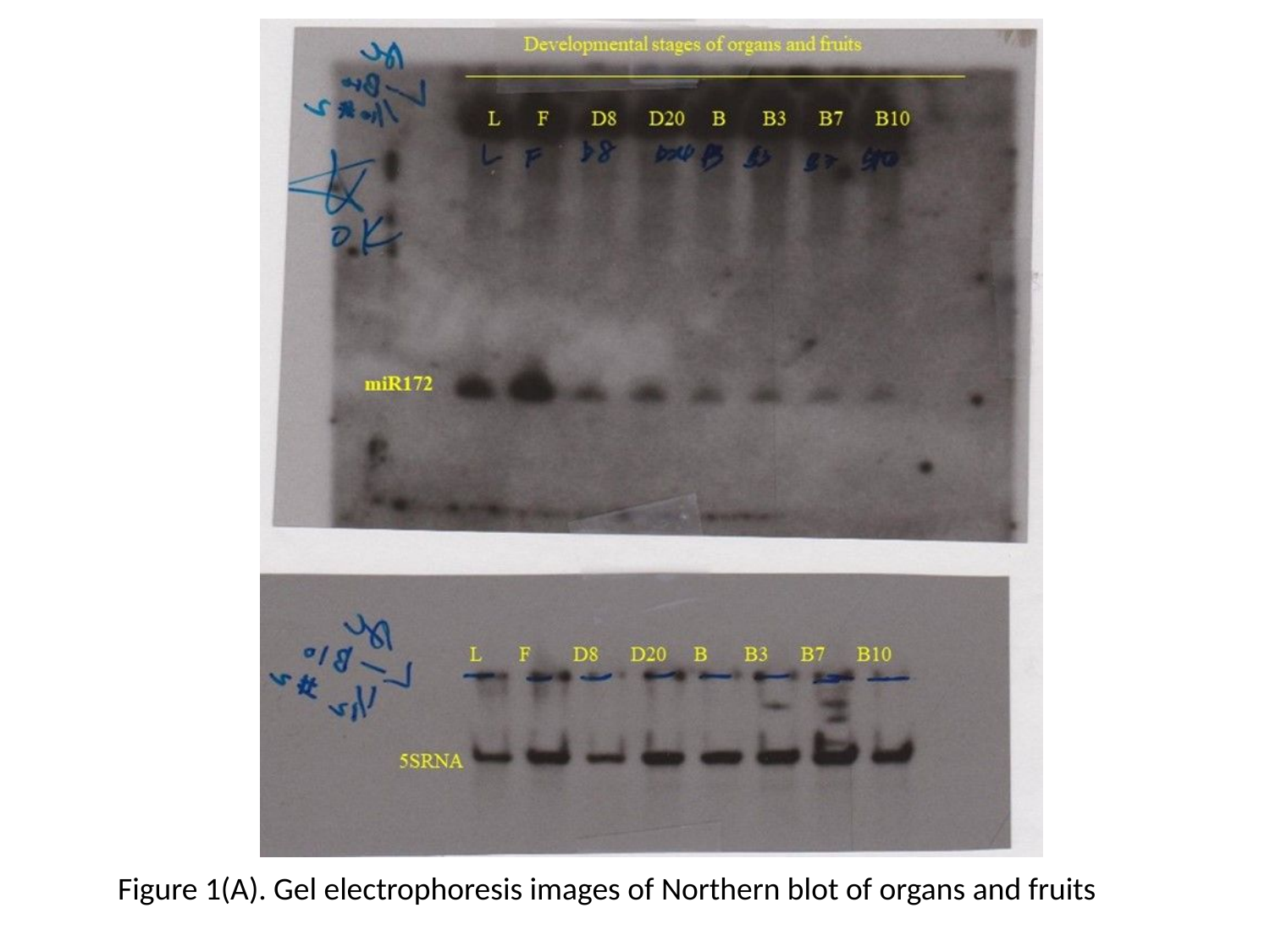

Figure 1(A). Gel electrophoresis images of Northern blot of organs and fruits

## Slide 2
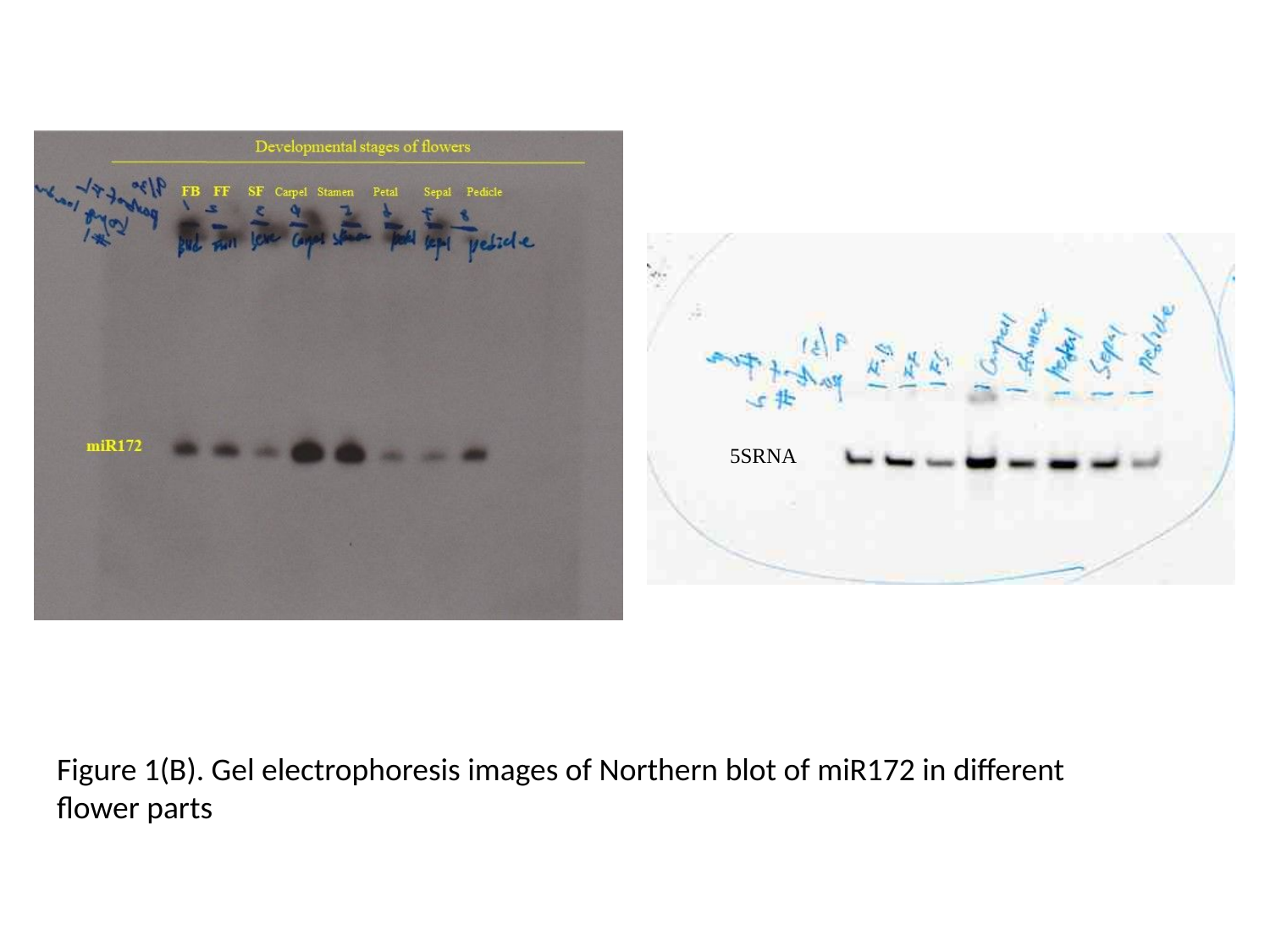

5SRNA
Figure 1(B). Gel electrophoresis images of Northern blot of miR172 in different flower parts

## Slide 3
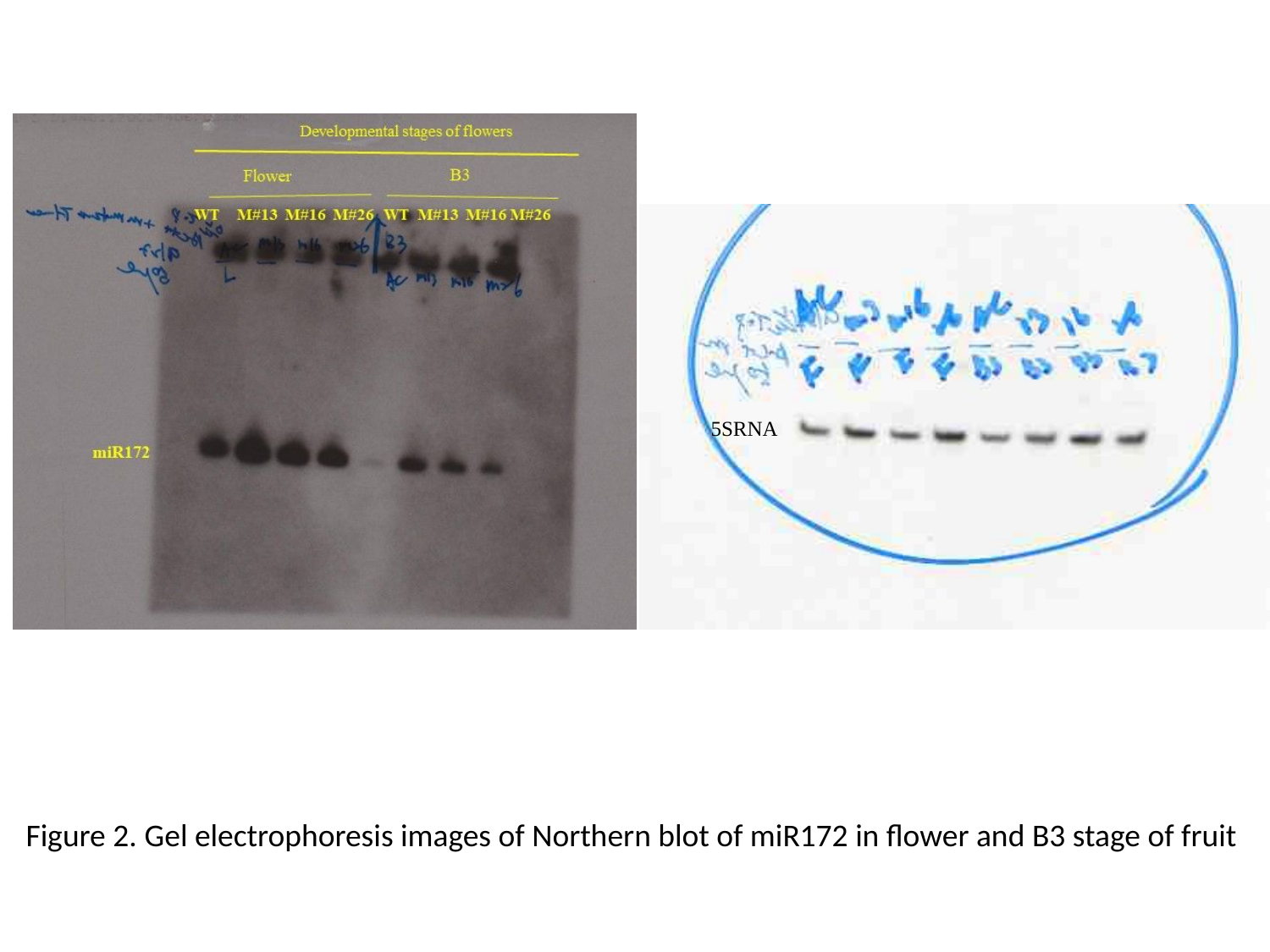

5SRNA
Figure 2. Gel electrophoresis images of Northern blot of miR172 in flower and B3 stage of fruit

## Slide 4
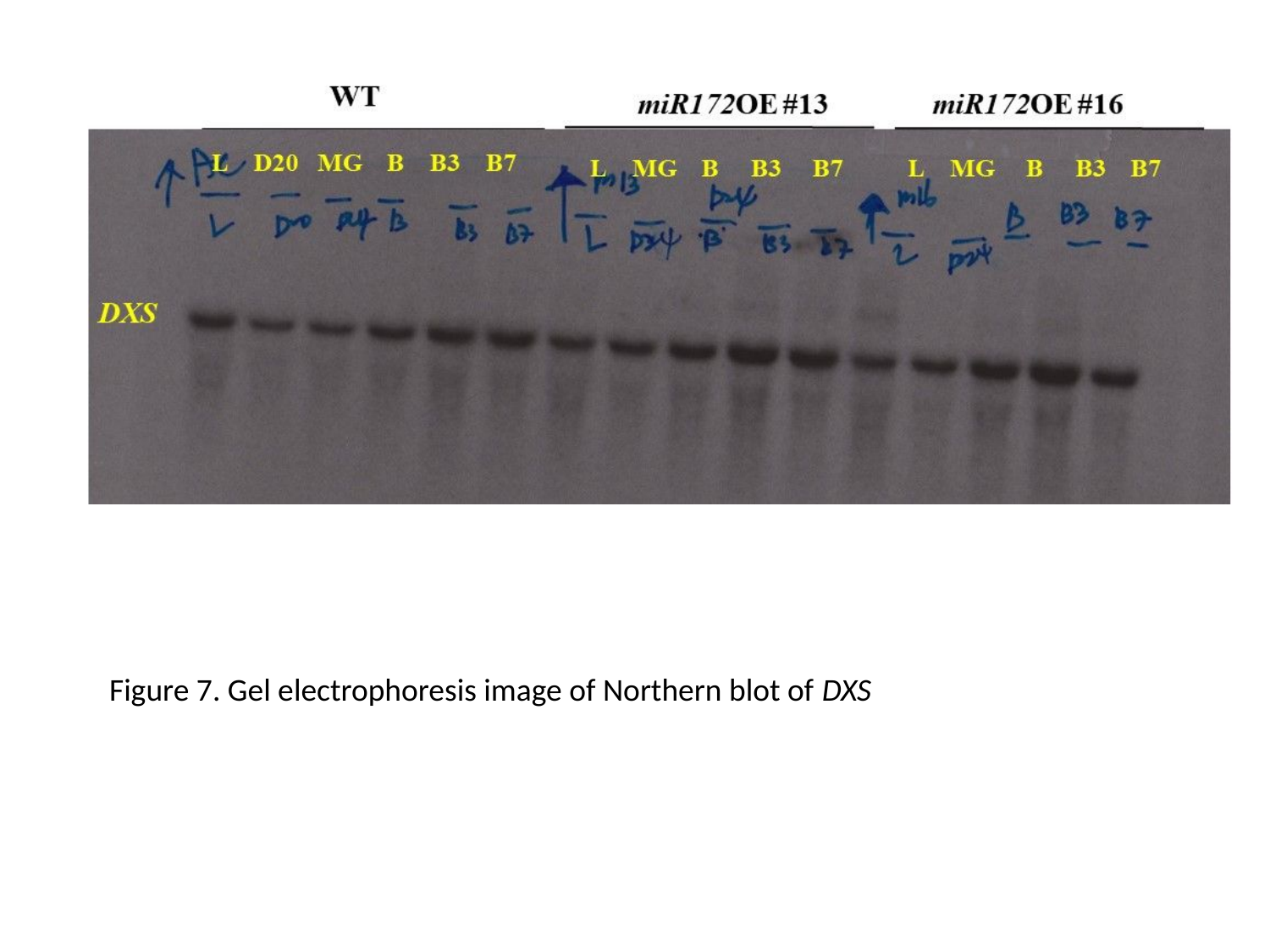

Figure 7. Gel electrophoresis image of Northern blot of DXS

## Slide 5
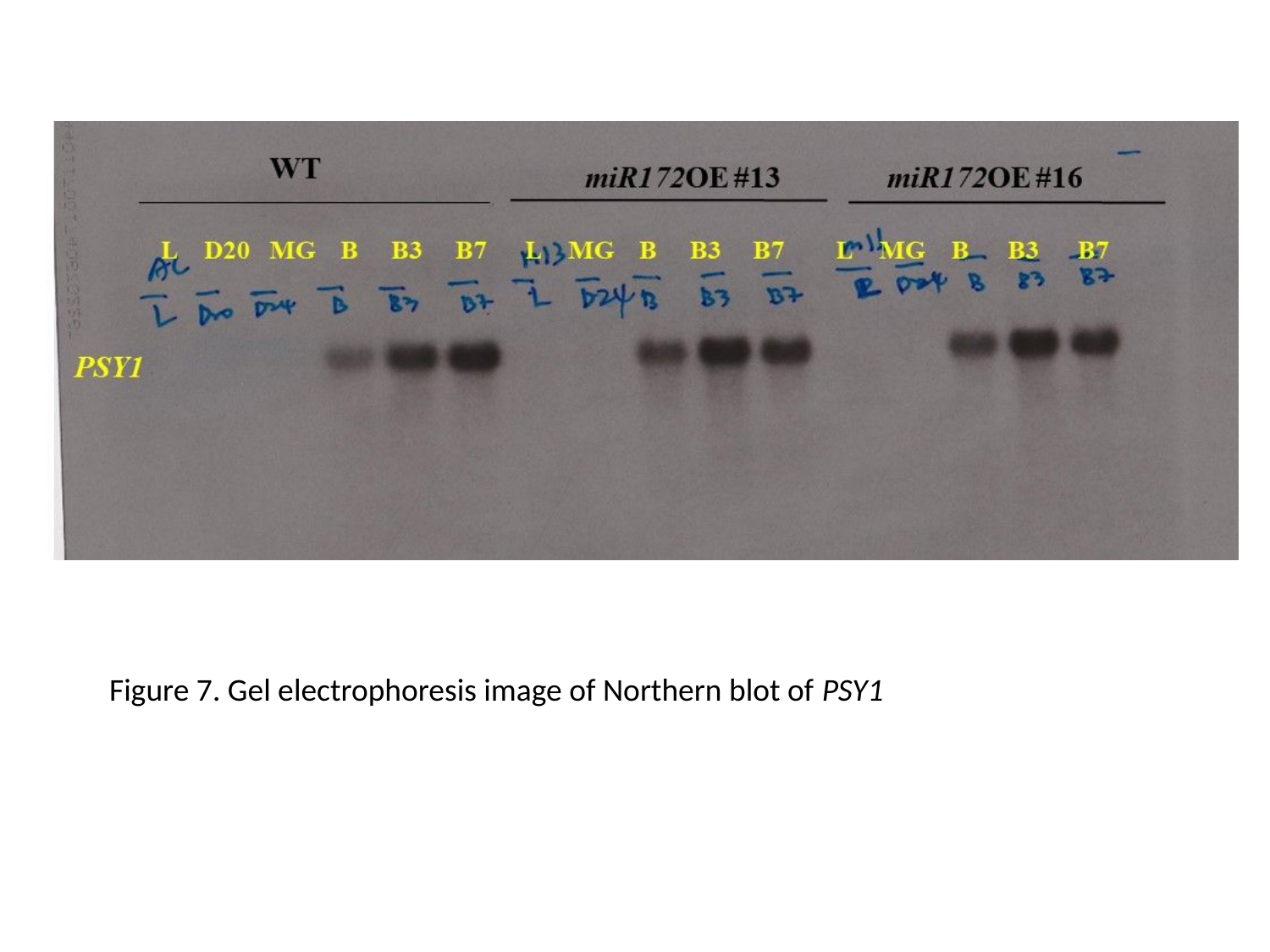

Figure 7. Gel electrophoresis image of Northern blot of PSY1

## Slide 6
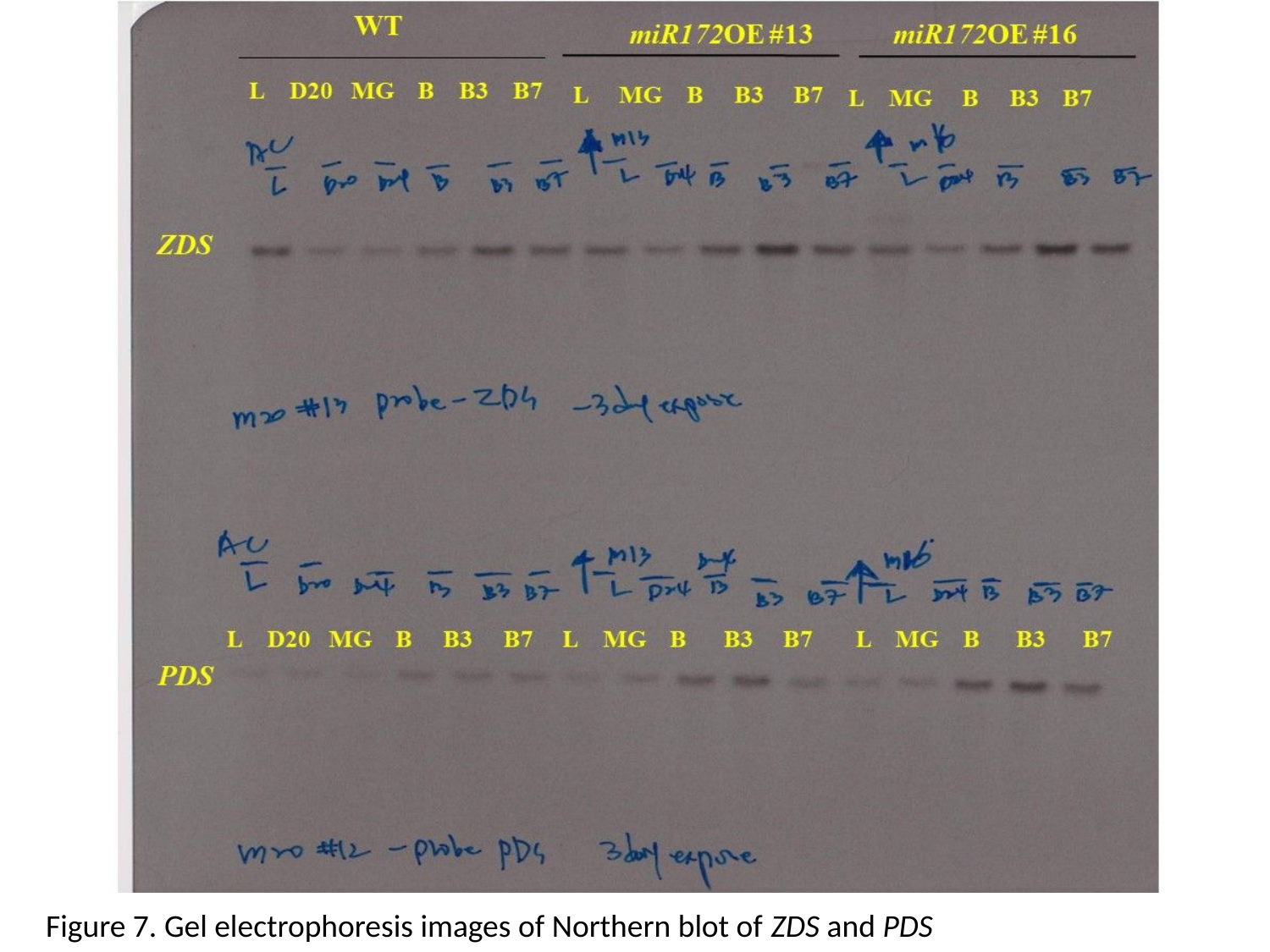

Figure 7. Gel electrophoresis images of Northern blot of ZDS and PDS

## Slide 7
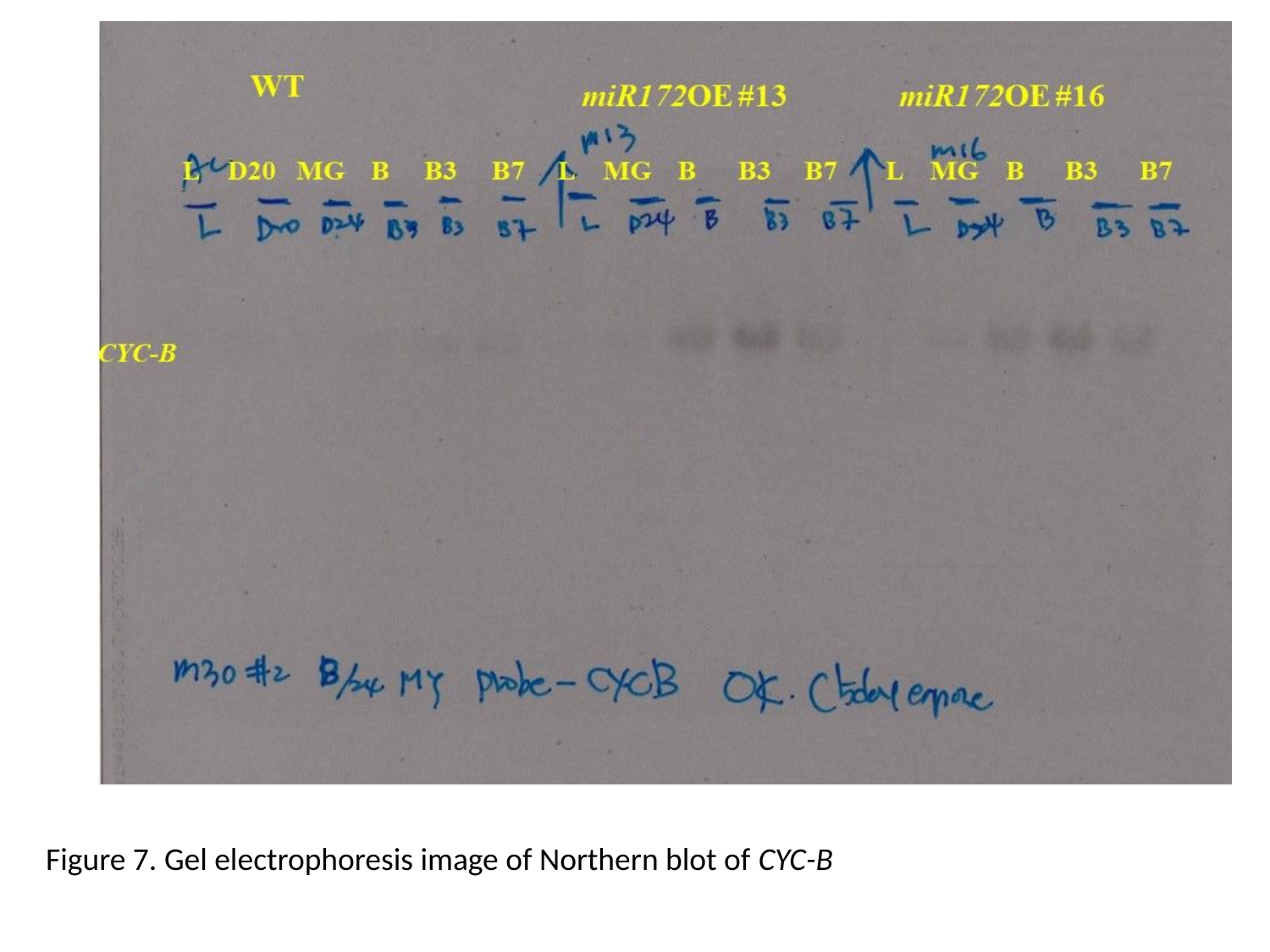

Figure 7. Gel electrophoresis image of Northern blot of CYC-B

## Slide 8
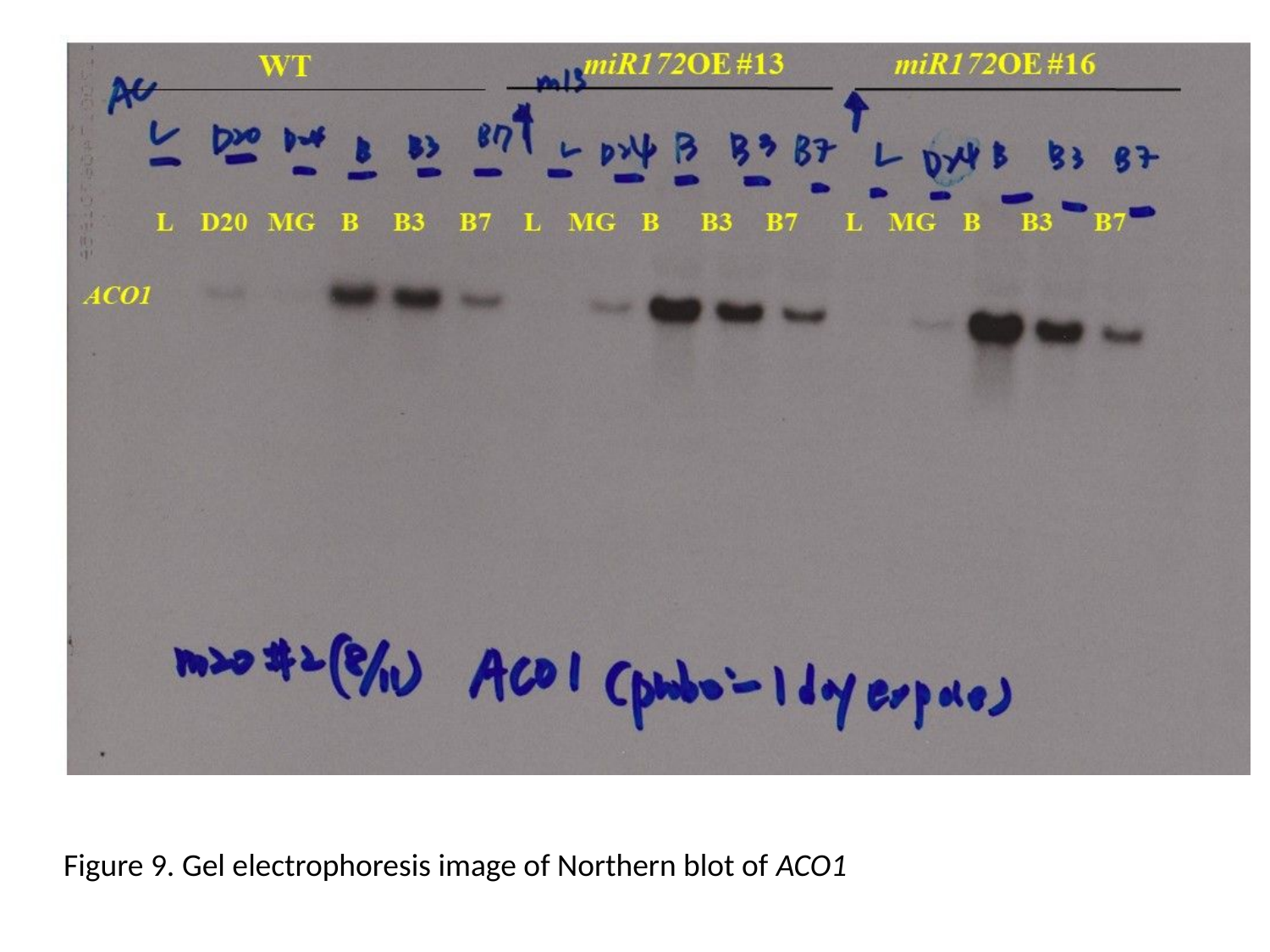

Figure 9. Gel electrophoresis image of Northern blot of ACO1

## Slide 9
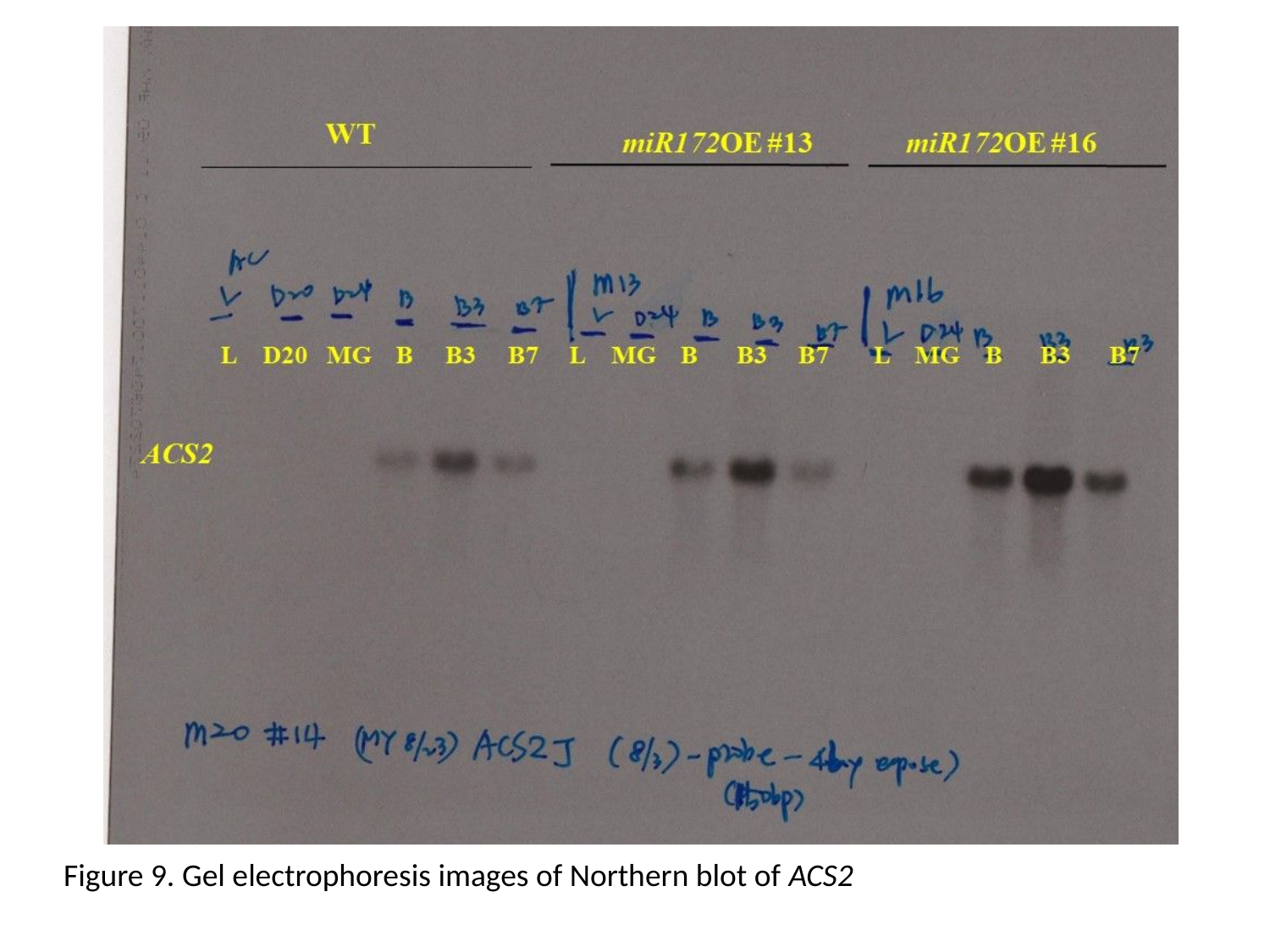

Figure 9. Gel electrophoresis images of Northern blot of ACS2

## Slide 10
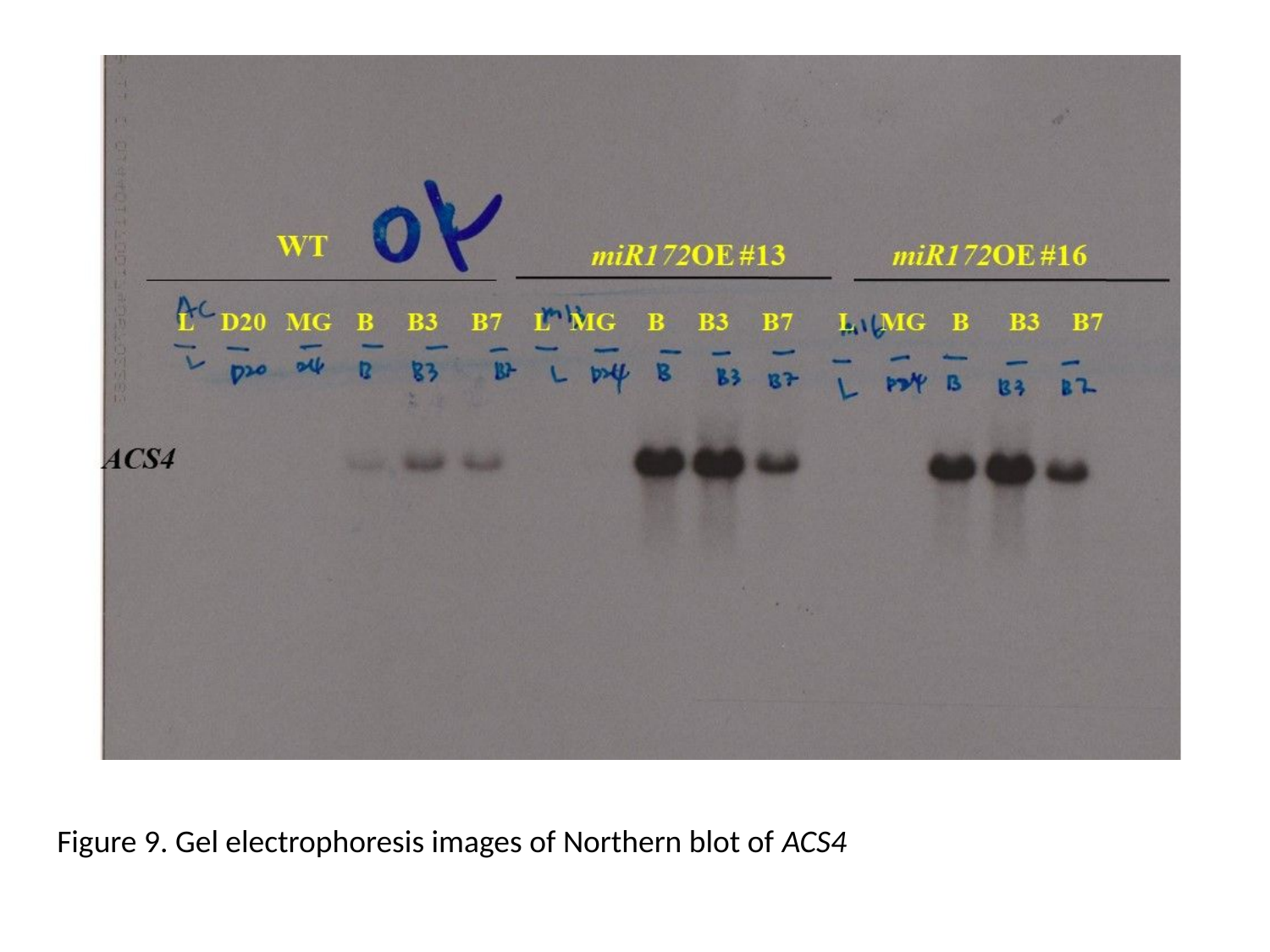

Figure 9. Gel electrophoresis images of Northern blot of ACS4

## Slide 11
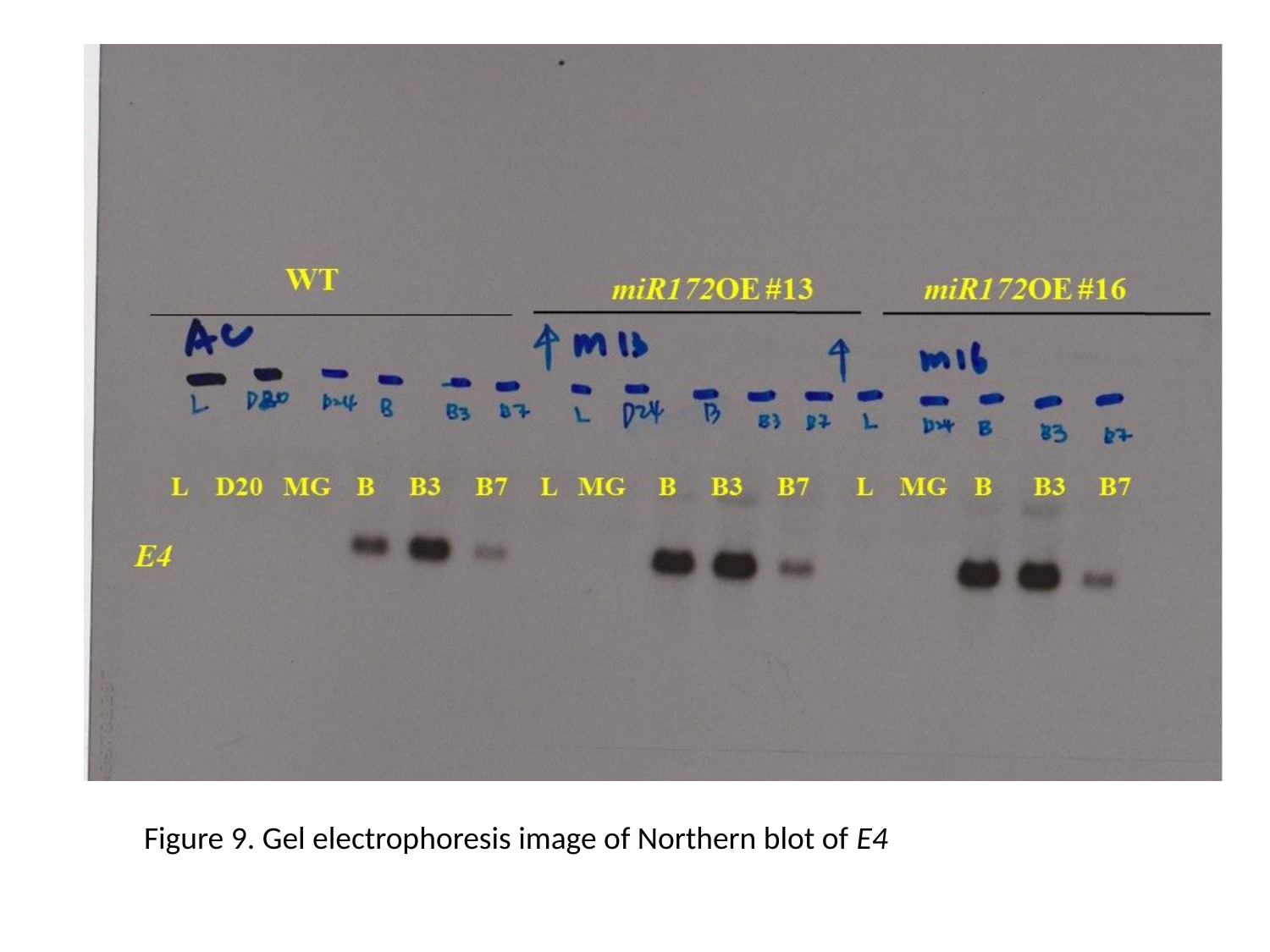

Figure 9. Gel electrophoresis image of Northern blot of E4

## Slide 12
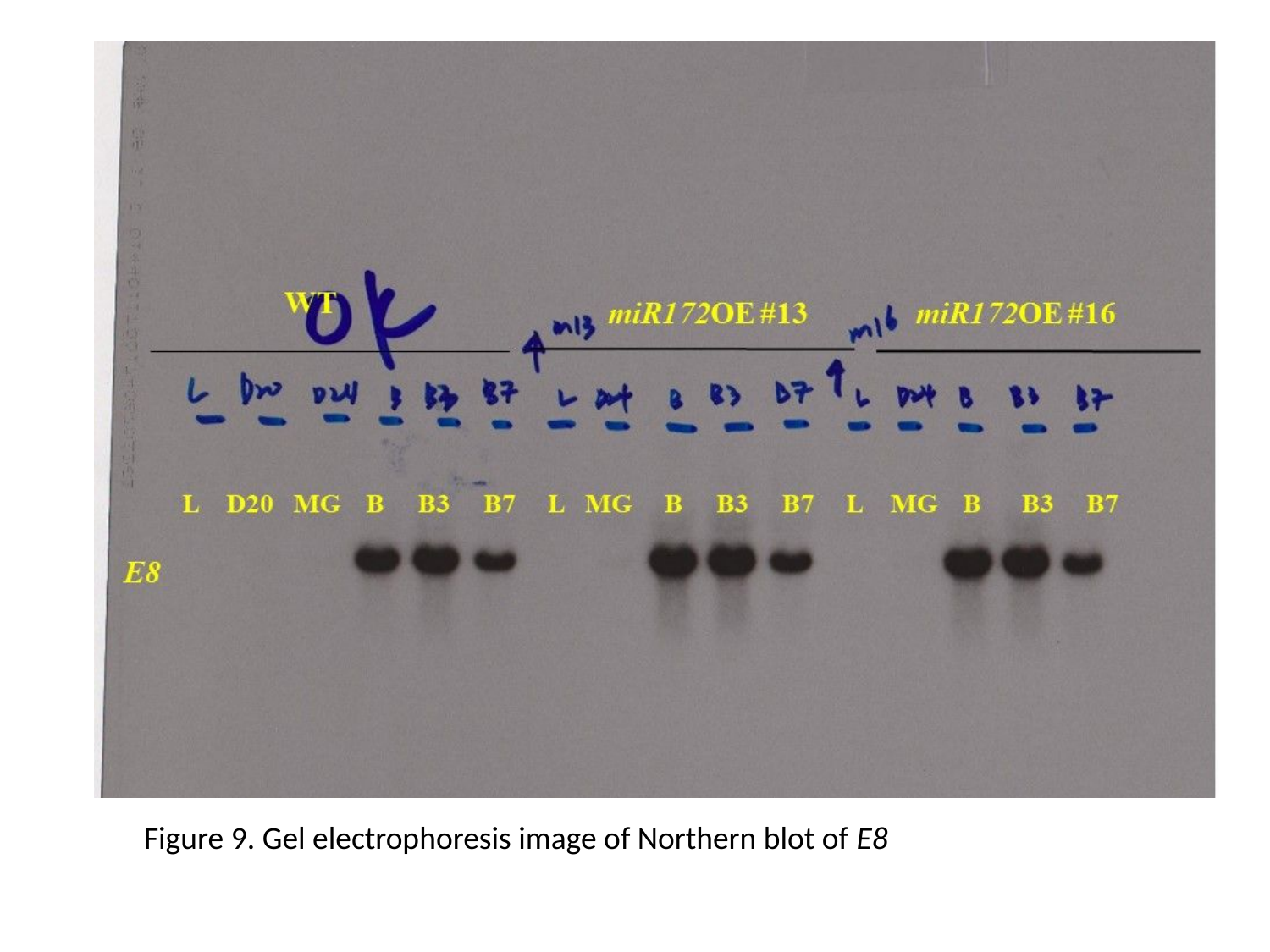

Figure 9. Gel electrophoresis image of Northern blot of E8

## Slide 13
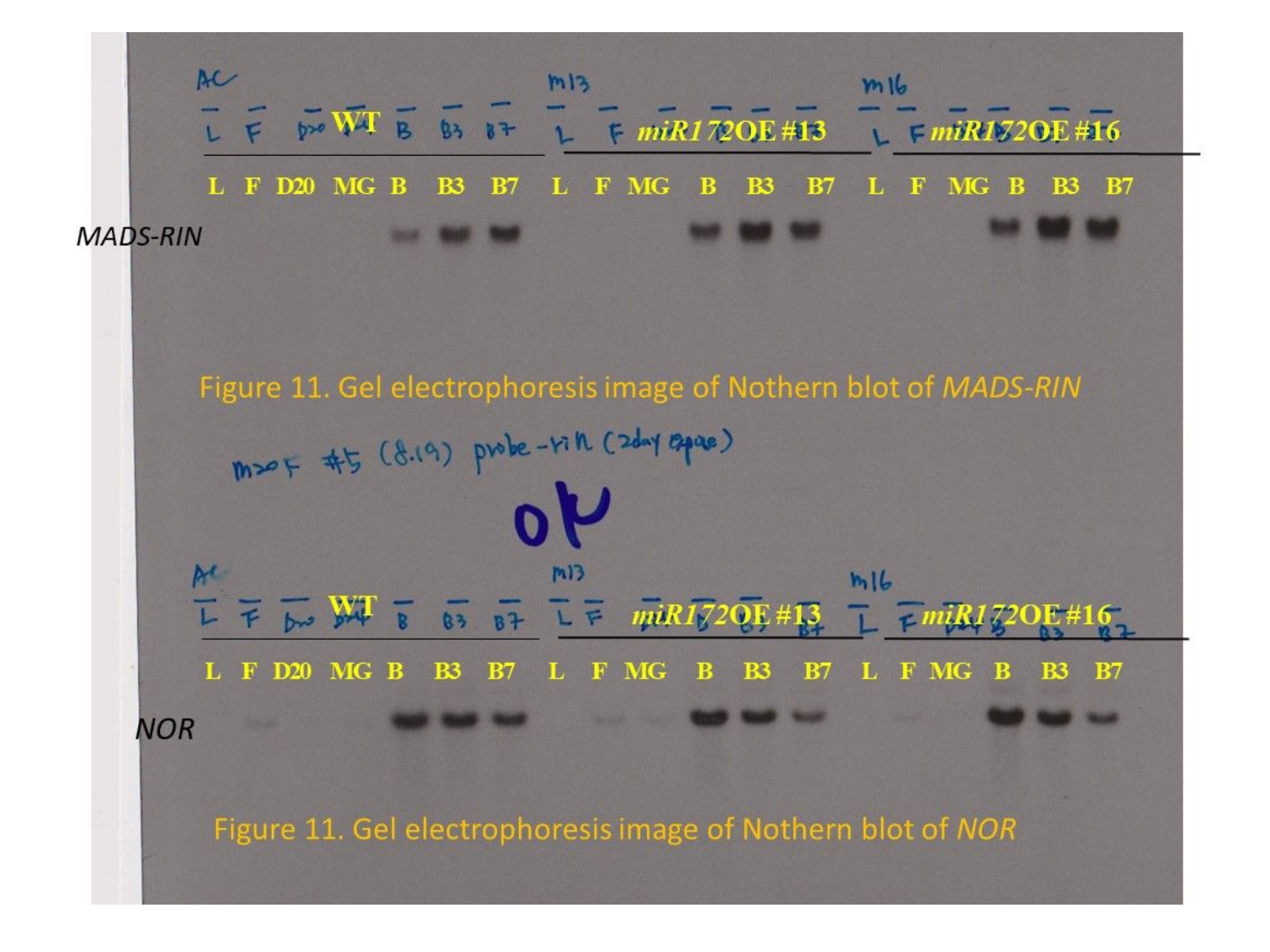

## Slide 14
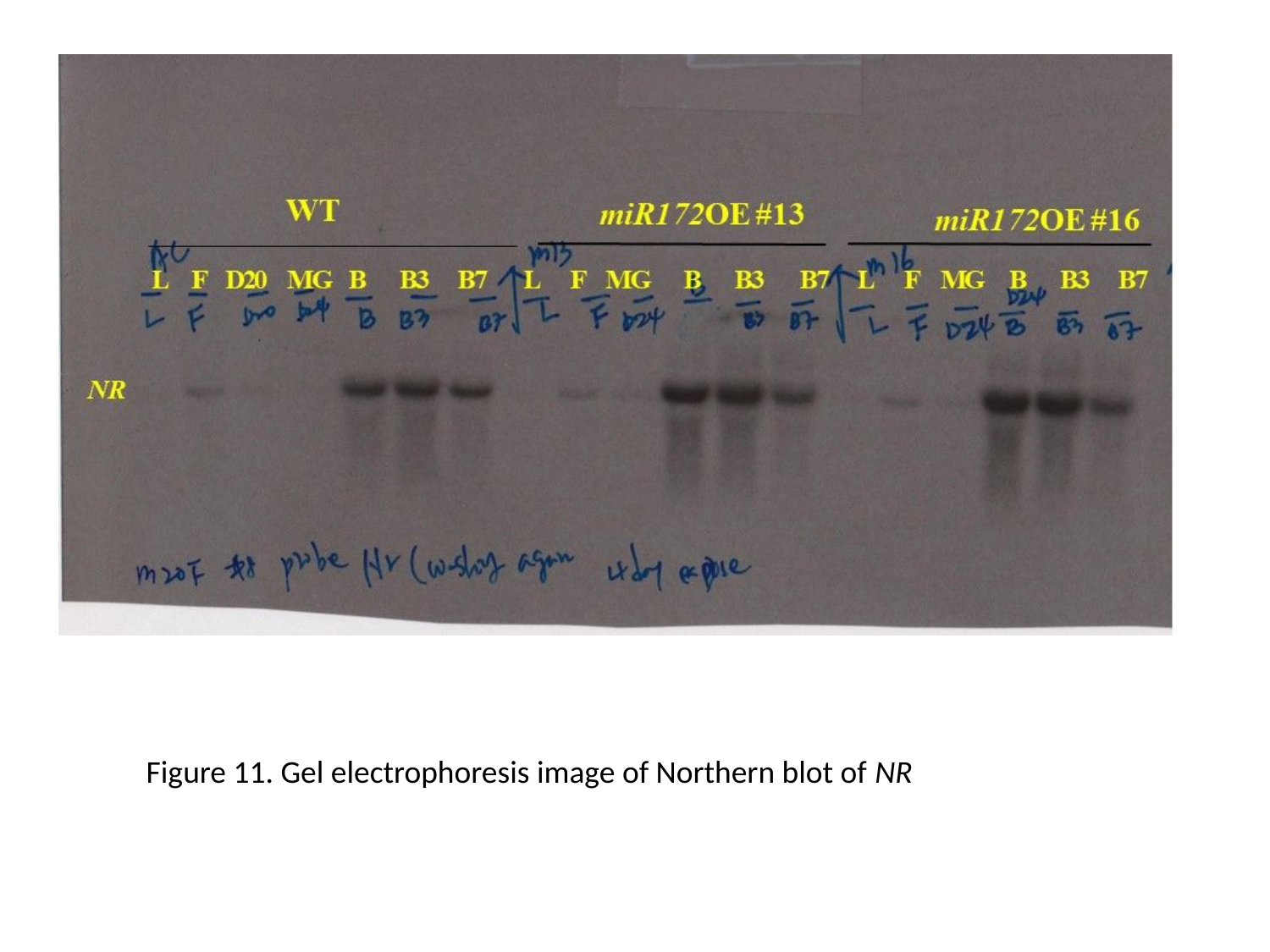

Figure 11. Gel electrophoresis image of Northern blot of NR

## Slide 15
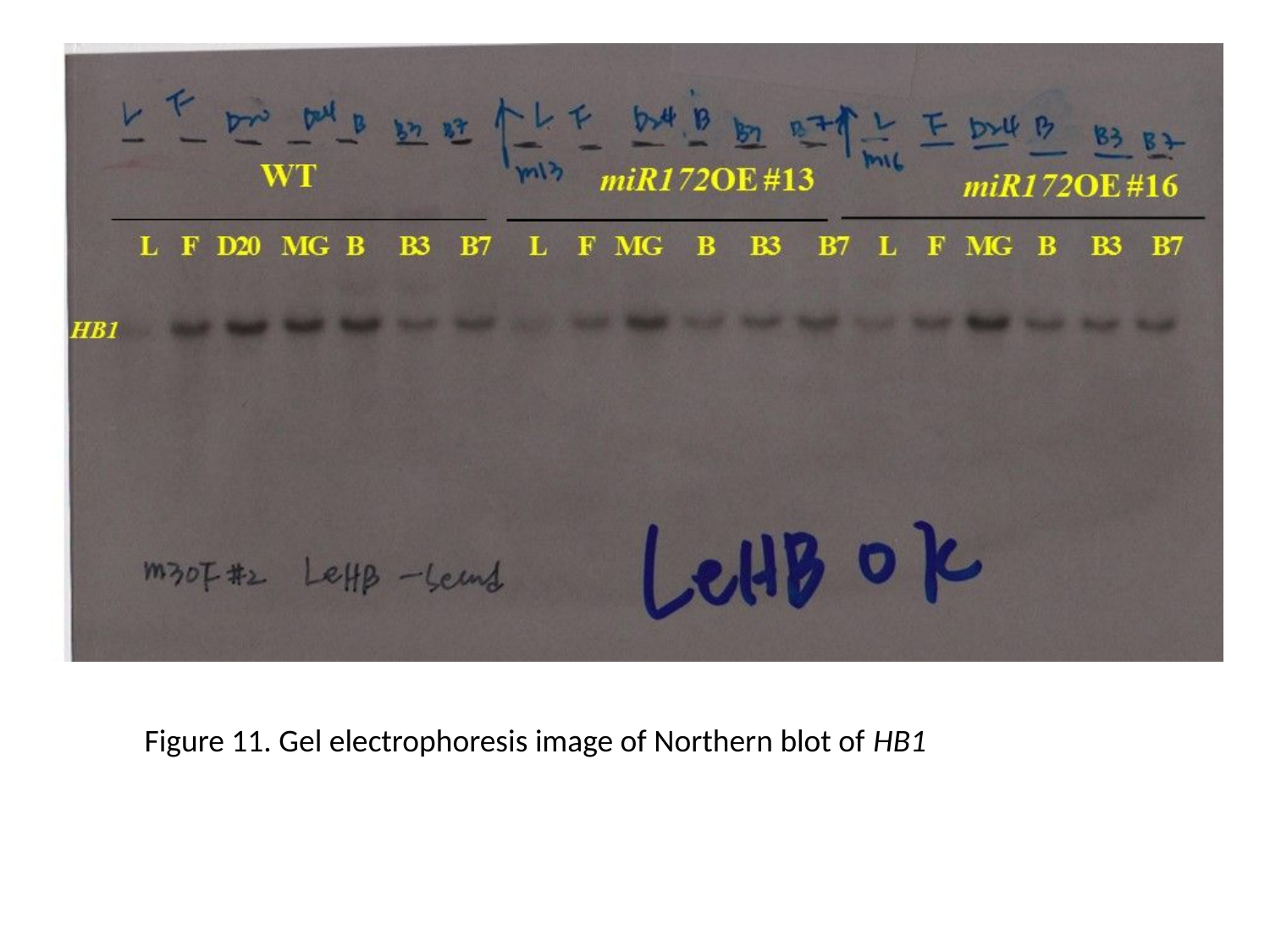

Figure 11. Gel electrophoresis image of Northern blot of HB1

## Slide 16
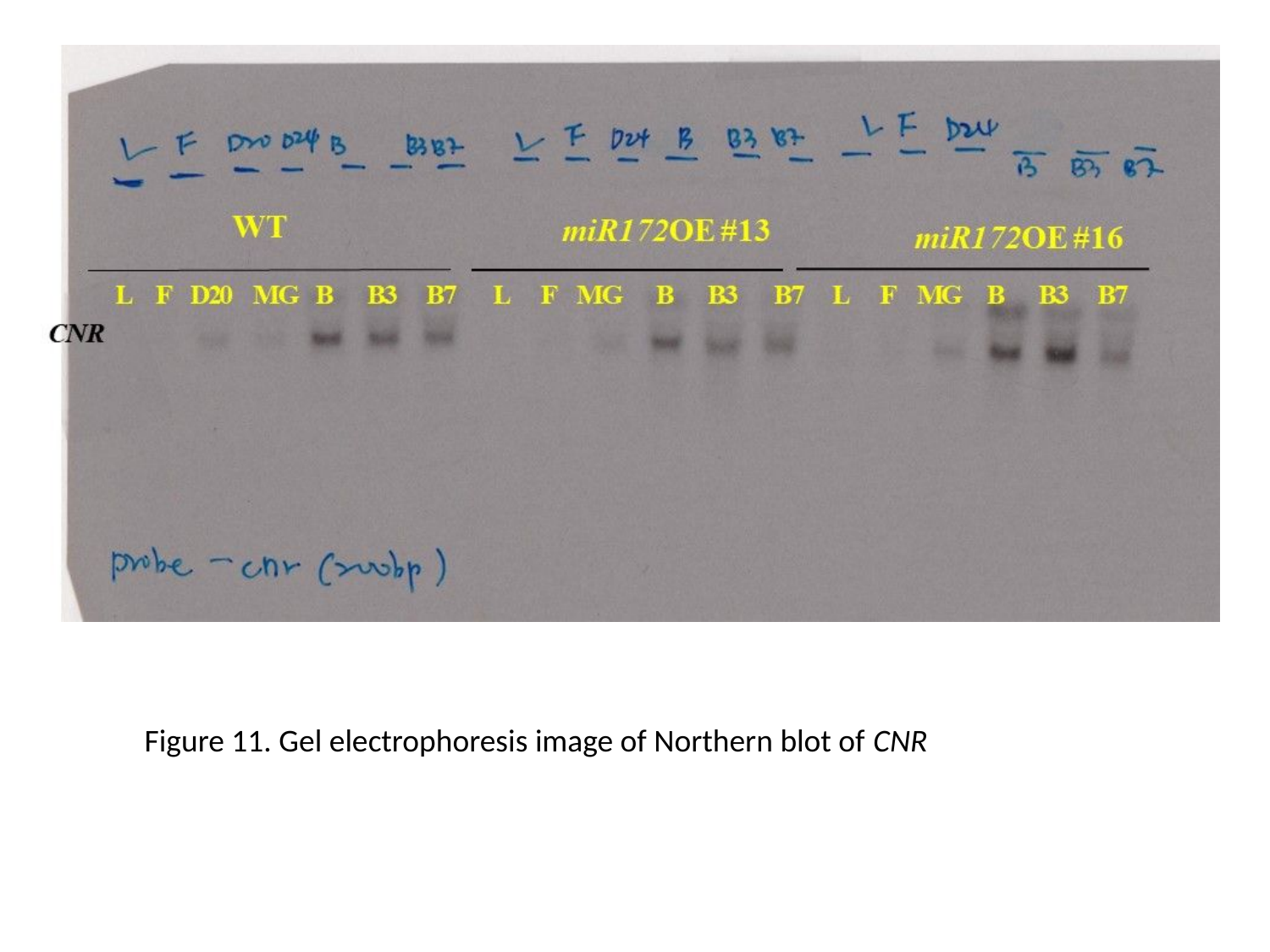

Figure 11. Gel electrophoresis image of Northern blot of CNR

## Slide 17
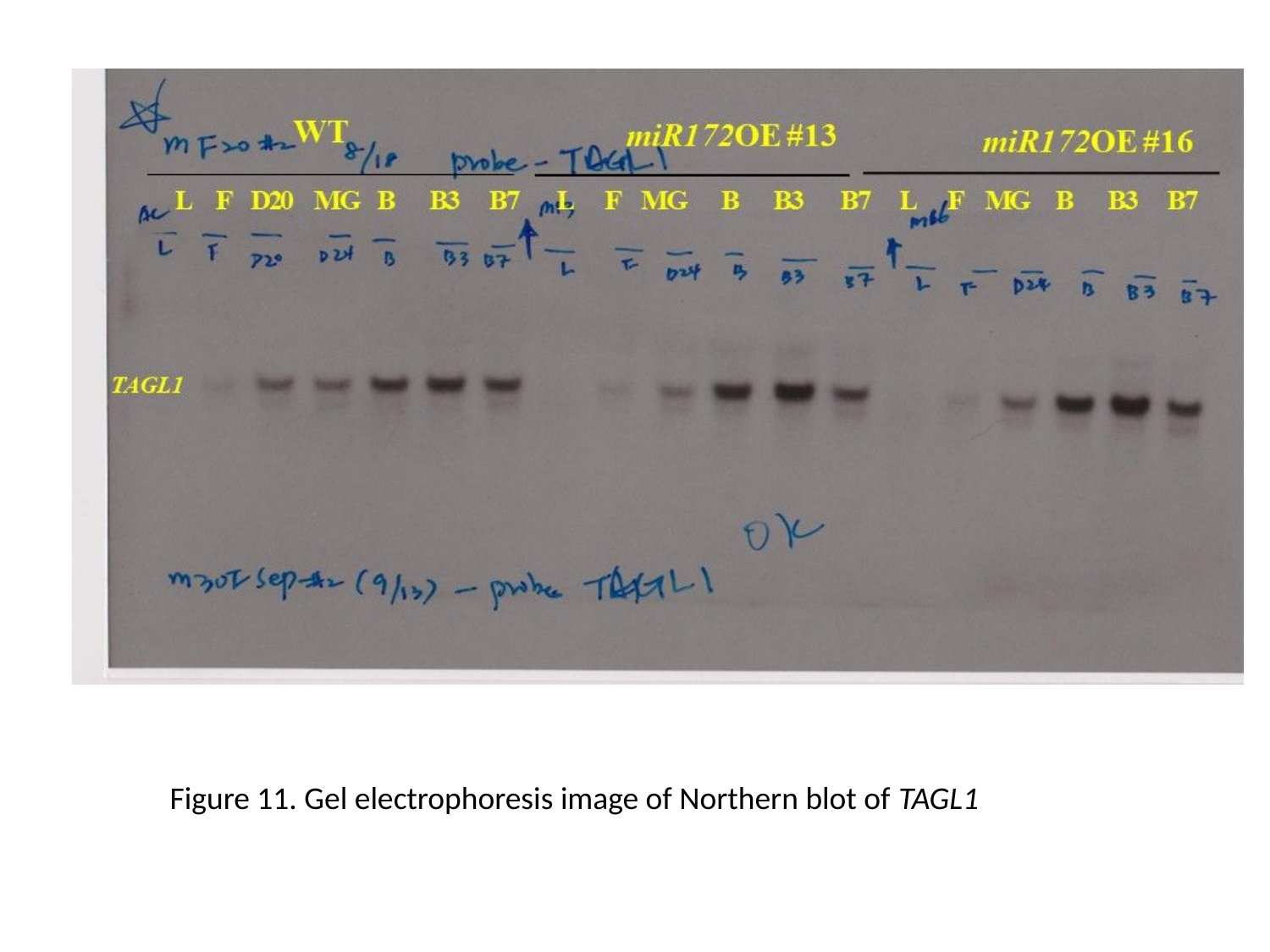

Figure 11. Gel electrophoresis image of Northern blot of TAGL1

## Slide 18
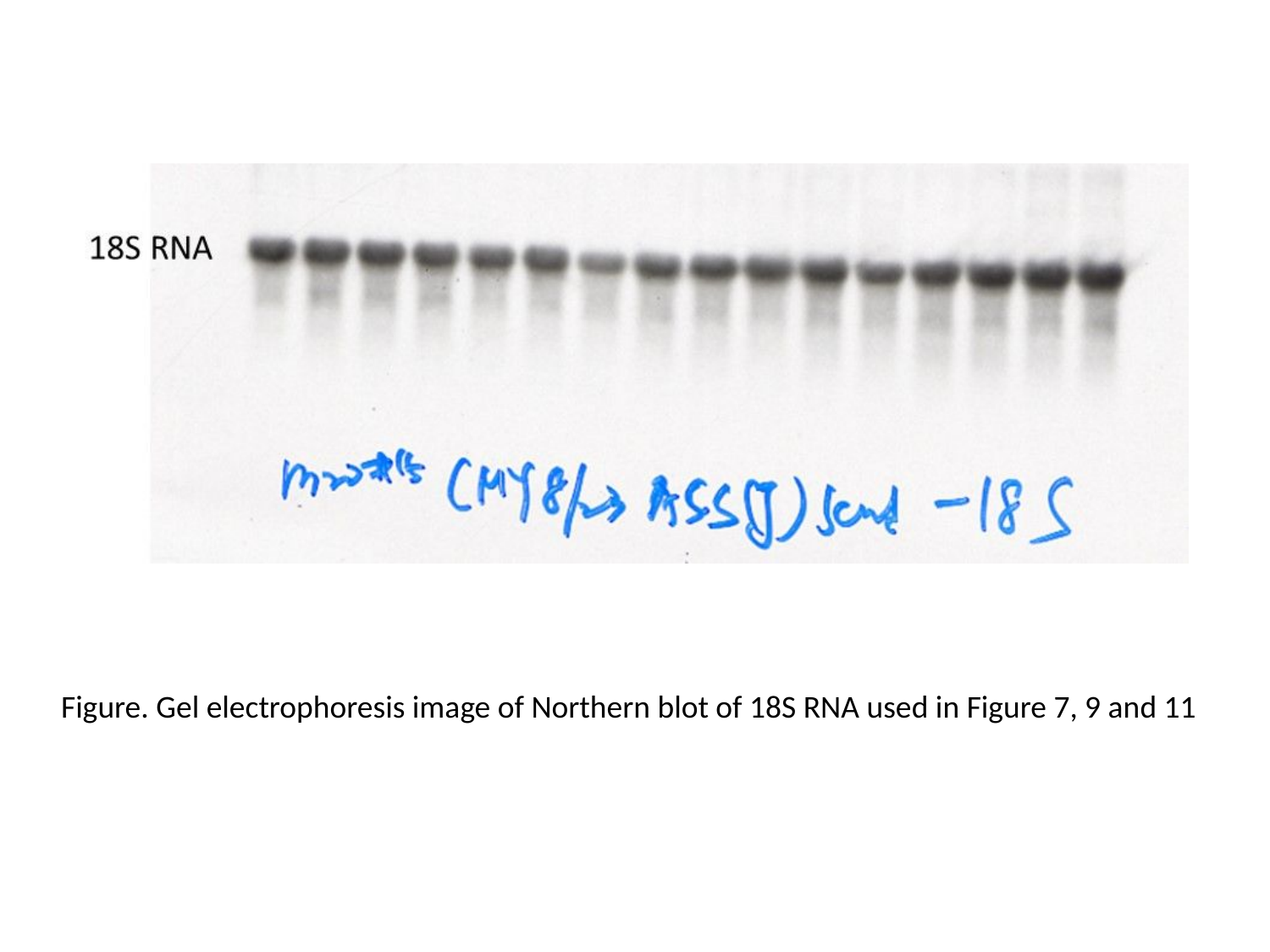

Figure. Gel electrophoresis image of Northern blot of 18S RNA used in Figure 7, 9 and 11

## Slide 19
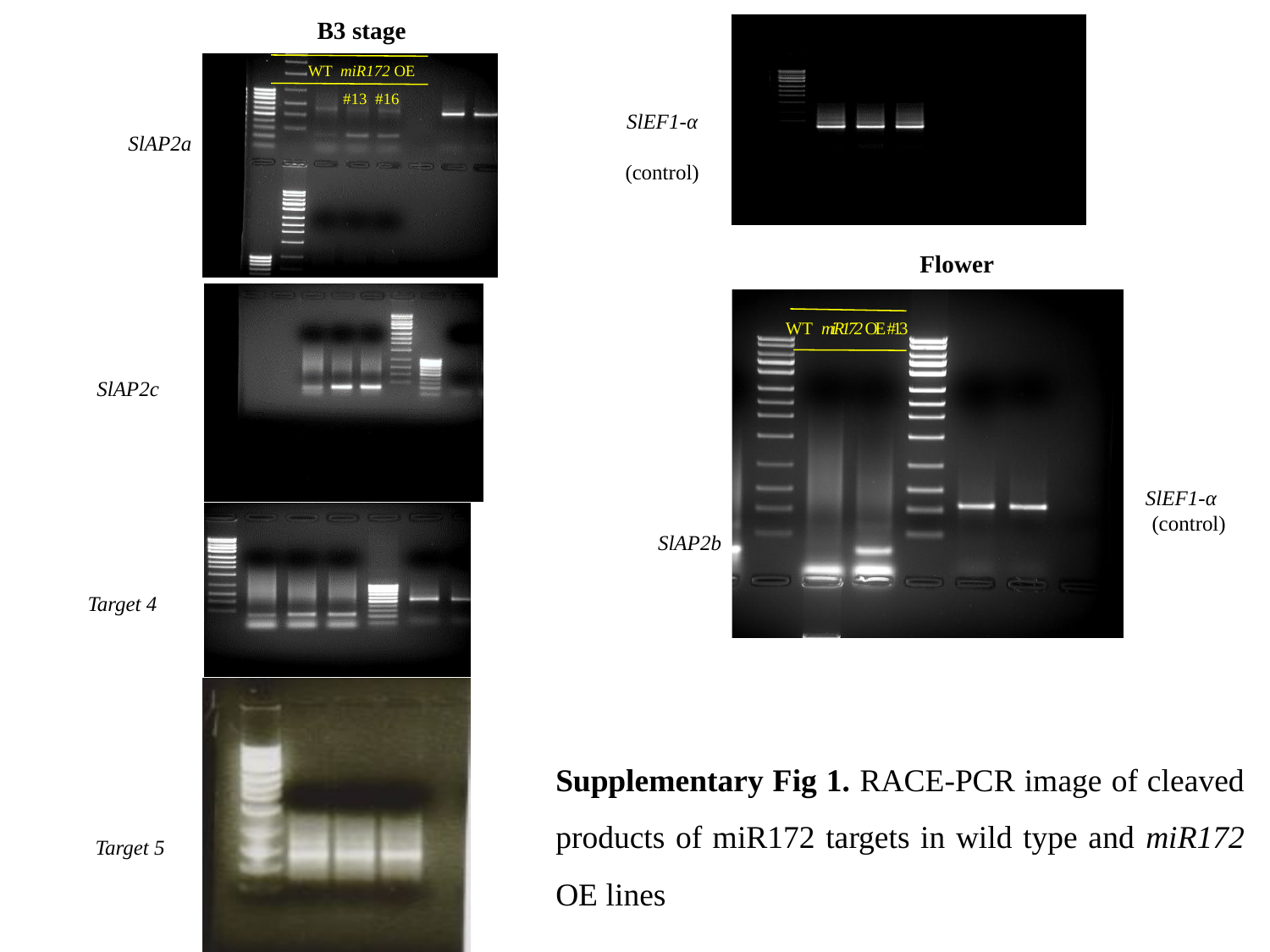

B3 stage
SlEF1-α
 (control)
WT miR172 OE
#13 #16
SlAP2a
Flower
WT miR172 OE #13
SlEF1-α
 (control)
SlAP2b
SlAP2c
Target 4
Target 5
Supplementary Fig 1. RACE-PCR image of cleaved products of miR172 targets in wild type and miR172 OE lines

## Slide 20
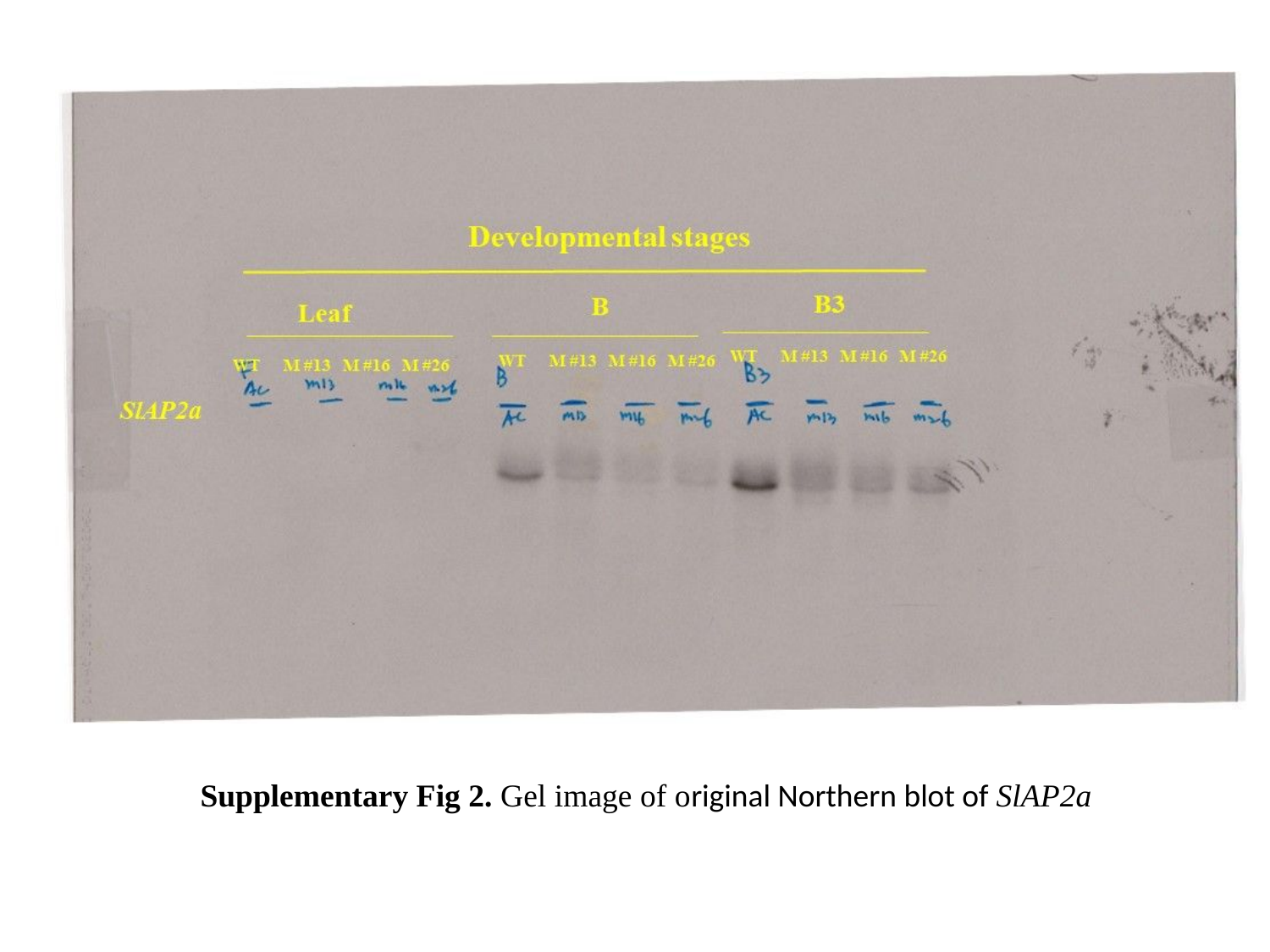

Supplementary Fig 2. Gel image of original Northern blot of SlAP2a

## Slide 21
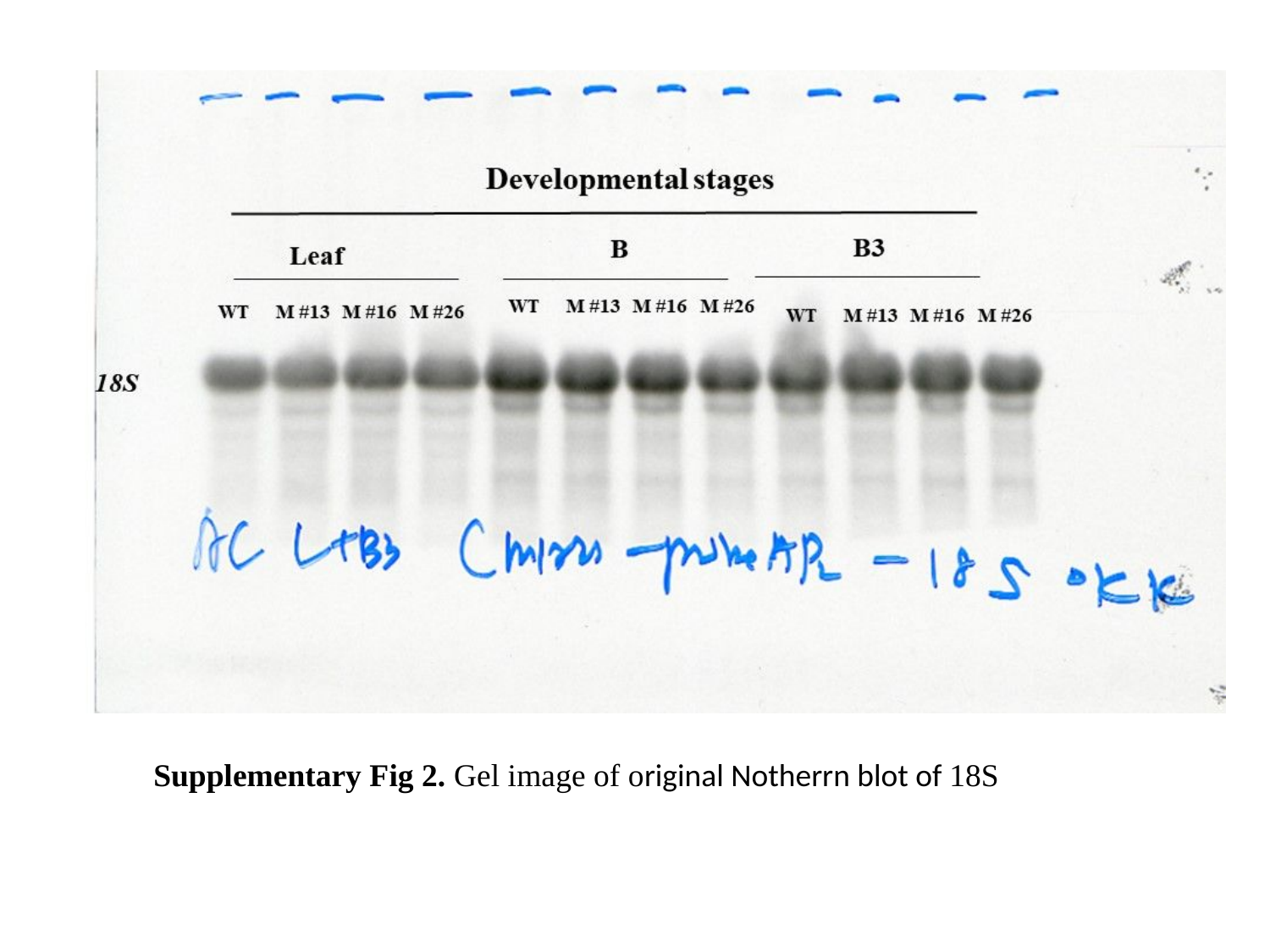

Supplementary Fig 2. Gel image of original Notherrn blot of 18S

## Slide 22
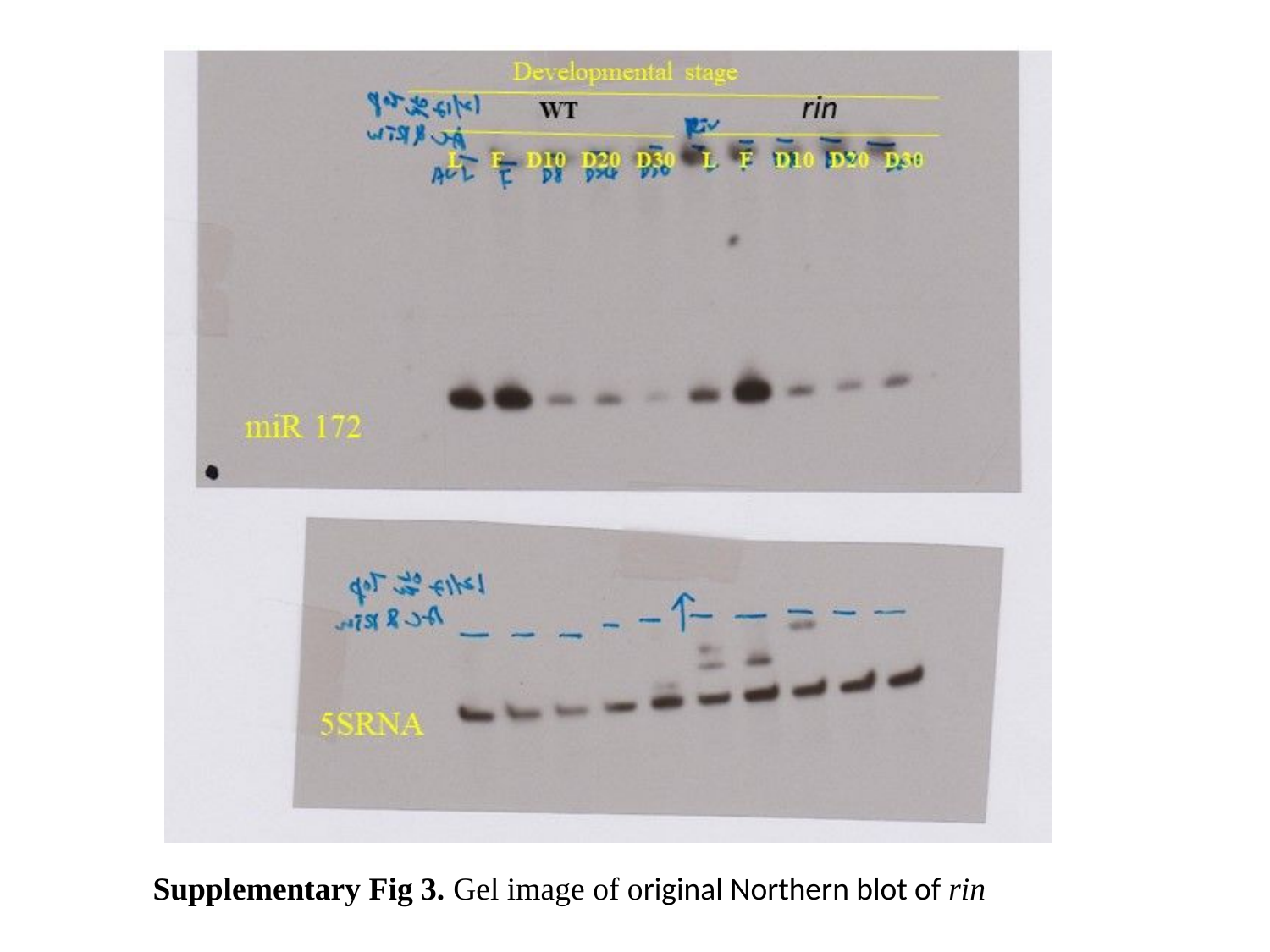

Supplementary Fig 3. Gel image of original Northern blot of rin

## Slide 23
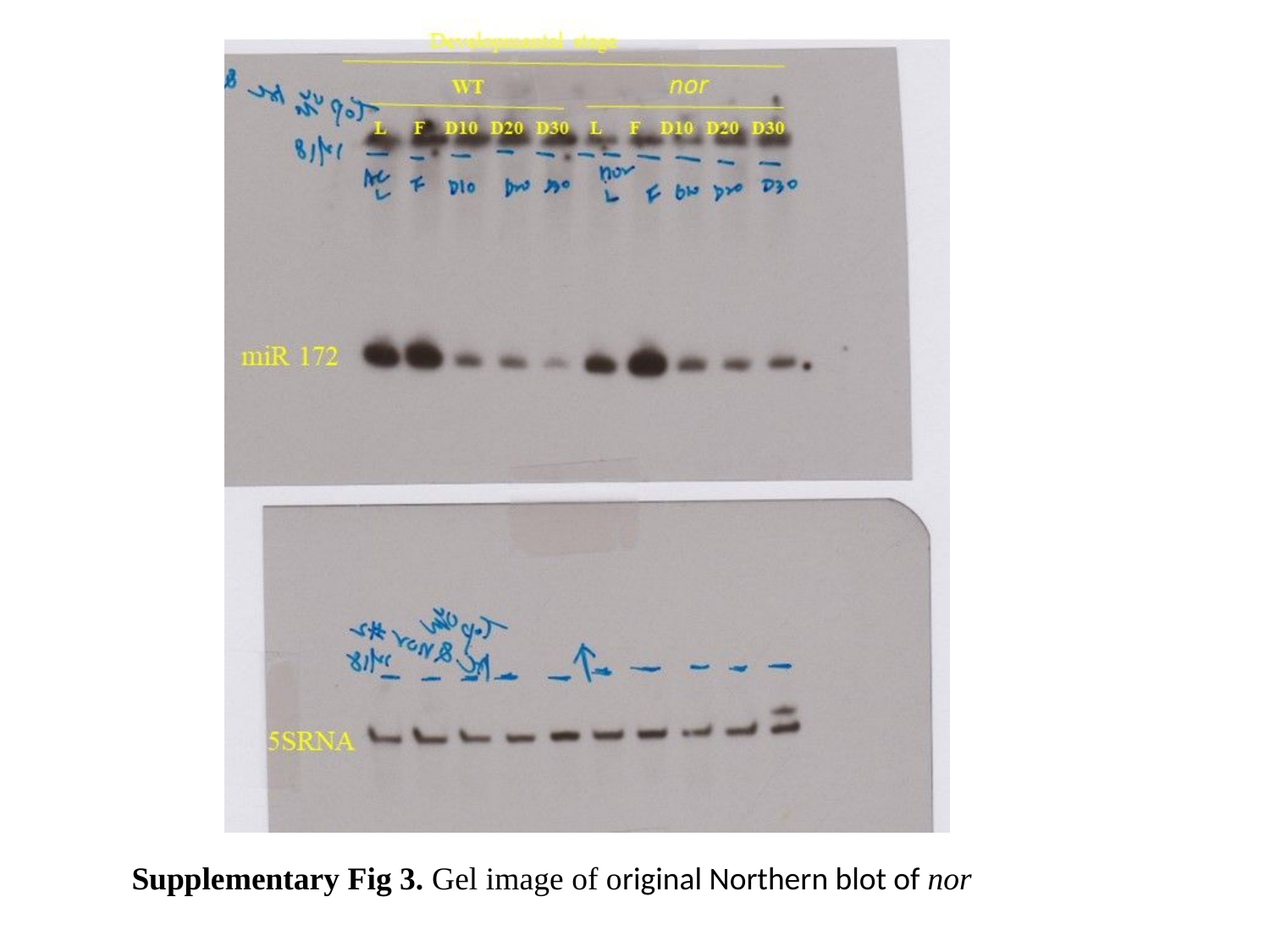

Supplementary Fig 3. Gel image of original Northern blot of nor

## Slide 24
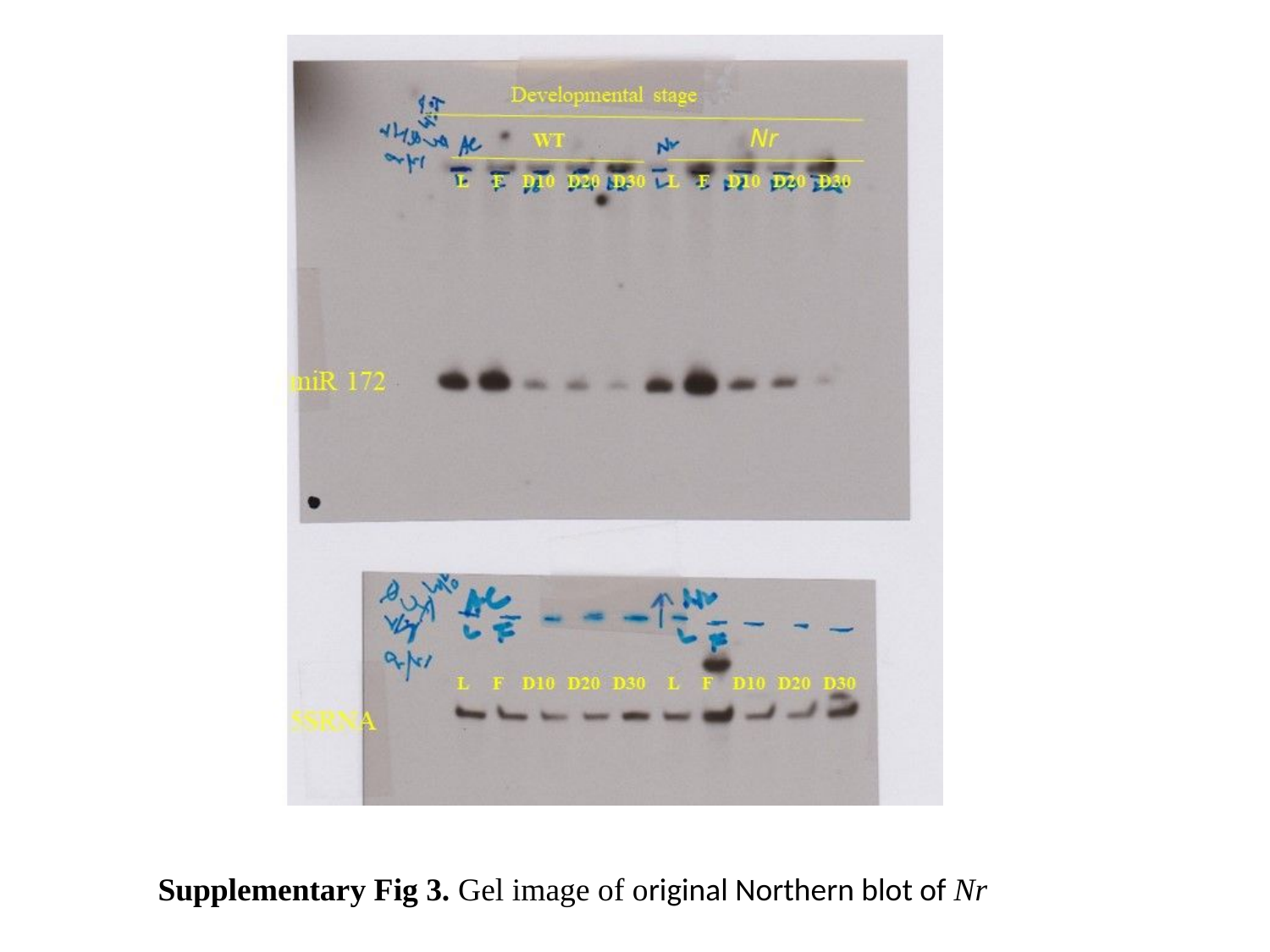

Supplementary Fig 3. Gel image of original Northern blot of Nr

## Slide 25
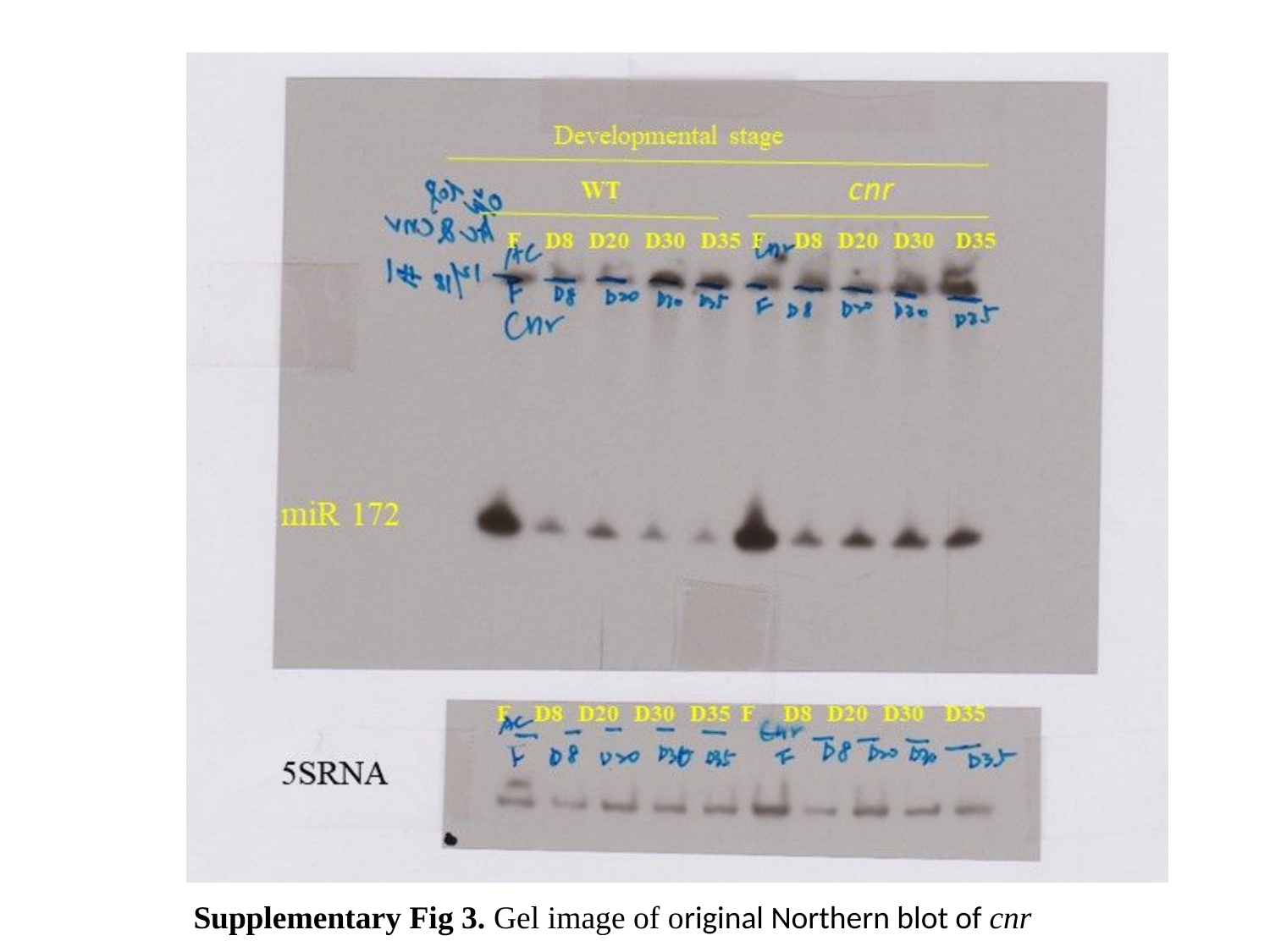

Supplementary Fig 3. Gel image of original Northern blot of cnr
